# circPDE5A regulates prostate cancer metastasis via controlling WTAP-dependent N6-methyladenisine methylation of EIF3C mRNA

**DOI:** 10.1186/s13046-022-02391-5

**Published:** 2022-06-02

**Authors:** Lifeng Ding, Ruyue Wang, Qiming Zheng, Danyang Shen, Huan Wang, Zeyi Lu, Wenqin Luo, Haiyun Xie, Liangliang Ren, Minxiao Jiang, Chenhao Yu, Zhenwei Zhou, Yudong Lin, Haohua Lu, Dingwei Xue, Wenjing Su, Liqun Xia, Jochen Neuhaus, Sheng Cheng, Gonghui Li

**Affiliations:** 1 Department of Urology, Sir Run Run Shaw Hospital, Zhejiang University School of Medicine, Hangzhou, 310016 China; 2 Department of General Surgery, The First Affiliated Hospital of Soochow University, Suzhou, 215006 China; 3 Life Sciences Institute, Zhejiang University, 866 Yuhangtang Road, Hangzhou, 310058 China; 4 Department of Urology, Research Laboratory, University Leipzig, D-04103 Leipzig, Germany

**Keywords:** Prostate cancer, Metastasis, circPDE5A, WTAP, EIF3C

## Abstract

**Background:**

Circular RNA (circRNA) is a novel class noncoding RNA (ncRNA) that plays a critical role in various cancers, including prostate cancer (PCa). However, the clinical significance, biological function, and molecular mechanisms of circRNAs in prostate cancer remain to be elucidated.

**Methods:**

A circRNA array was performed to identified the differentially expressed circRNAs. circPDE5A was identified as a novel circRNA which downregulated in clinical samples. Functionally, the in vitro and in vivo assays were applied to explore the role of circPDE5A in PCa metastasis. Mechanistically, the interaction between circPDE5A and WTAP was verified using RNA pulldown followed by mass spectrometry, RNA Immunoprecipitation (RIP) assays. m^6^A methylated RNA immunoprecipitation sequencing (MeRIP-seq) was then used to identified the downstream target of circPDE5A. Chromatin immunoprecipitation assay (ChIP) and dual-luciferase reporter assay were used to identified transcriptional factor which regulated circPDE5A expression.

**Results:**

circPDE5A was identified downregulated in PCa tissues compared to adjacent normal tissue and was negatively correlated with gleason score of PCa patients. circPDE5A inhibits PCa cells migration and invasion both in vitro and in vivo. circPDE5A blocks the WTAP-dependent N6-methyladenisine (m^6^A) methylation of eukaryotic translation initiation factor 3c (EIF3C) mRNA by forming the circPDE5A-WTAP complex, and finally disrupts the translation of EIF3C. Moreover, the circPDE5A-dependent decrease in EIF3C expression inactivates the MAPK pathway and then restrains PCa progression.

**Conclusions:**

Our findings demonstrate that FOXO4-mediated upregulation of circPDE5A controls PCa metastasis via the circPDE5A-WTAP-EIF3C-MAPK signaling pathway and could serve as a potential therapeutic targer for PCa.

**Supplementary Information:**

The online version contains supplementary material available at 10.1186/s13046-022-02391-5.

## Introduction

Prostate cancer is the second commonly diagnosed cancer (13.5% of total cases) in man worldwide [1]. Although the advanced early detection and therapeutic strategies, many patients with prostate cancer still progress to the metastatic status [2, 3]. According to its sensitivity to androgen deprivation therapy (ADT), metastatic prostate cancer could be divided into metastatic hormone-sensitive prostate cancer (mHSPC) and metastatic castration-resistant prostate cancer (mCRPC) [4]. However, despite the great progression in the therapeutic drugs, such as enzalutamide, abiraterone, taxanes, radium-223, the disease will ultimately relapse and become resistant to these treatments [5, 6]. Thus, the additional therapeutic targets are needed to treat metastatic prostate cancer.

Nowadays, noncoding RNAs have played the vital roles in various physiological and pathological processes [7, 8]. Increasing evidence reveals that ncRNAs regulate cancer-related networks in transcriptional or post-transcriptional levels during tumorigenesis [9, 10]. circRNAs are newly recognized ncRNAs, essentially expressed in nearly all cells. However, emerging studies have demonstrated that a proportion of circRNAs are dysregulated in cancers, and their functions in tumorigenesis are still elusive. The most common function of circRNAs is to serve as miRNA sponges [11]. Moreover, circRNAs can interact with many RNA binding proteins (RBPs) to block or enhance protein function. In addition, although named as ncRNAs, certain circRNAs are reported translatable in a cap-independent manner [12, 13]. Some circRNAs are reportedly related to PCa initiation and progression [14, 15], but the detailed molecular mechanism of circRNAs in PCa is still lacking.

The RNA chemical modifications of RNAs are an efficient way of regulating RNA function. Among these RNA modifications, N6-methyladenosine modification is the most abundant and conserved RNA modification in mammalian cells [16]. m^6^A modification, which modified by writers, including METTL3, METTL14, and WTAP, and removed by erasers, including FTO and ALKBH5, is dynamic and reversible [17]. Also, m^6^A modification is recognized by readers, including IGF2BP1/2/3, YTHDC1/2/3, YTHDF1/2/3, and HNRNPA2B1, to exert various biological processes, such as RNA processing, nuclear export, mRNA translation and so on [18]. Accumulating evidences showed that m^6^A methyltransferases, WTAP, play the vital role in tumor progression. Chen et al. reported that WTAP facilitated osteosarcoma progression through regulating HMBOX1 mRNA stability in a m^6^A-dependent manner [19]. Another group showed that WTAP regulated m^6^A modification of lncRNA DIAPH1-AS1 to promote nasopharyngeal carcinoma progression [20]. However, the role of WTAP in prostate cancer is still poorly understood.

In this study, a circRNA array was performed in five paired samples of PCa, and circPDE5A was validated to be significantly downregulated in prostate cancer tissues. In vitro and in vivo assays showed that circPDE5A inhibited metastasis of PCa cells. We further revealed that circPDE5A could reduce the m^6^A modification level of EIF3C mRNA by interacting with WTAP and then inhibit the translation of EIF3C. Moreover, we found that FOXO4 and eIF4A3 could regulate the biogenesis of circPDE5A. Our study suggests that circPDE5A could be a potential diagnostic biomarker and an effective therapeutic target for PCa treatment.

## Methods and materials

### Clinical samples

All clinical prostate cancer samples were obtained with informed consent at Sir Run Run Shaw Hospital, School of Medicine, Zhejiang University. The Ethics Committee of Sir Run Run Shaw Hospital, School of Medicine, Zhejiang University approved this study. Detailed clinical characteristics of the patients are presented in Supplementary Table S2.

### Cell lines and cell culture

Human prostate cancer cells (LNCaP, C4-2B, 22Rv-1, DU145, and PC-3), and human normal prostate epithelial RWPE-1 cells were purchased from ATCC. LNCaP, C4-2B, 22Rv-1, DU145 and PC-3 cells were cultured in RPMI 1640 (Gibco) with 10% fetal bovine serum (Cellmax). RWPE-1 was cultured in Keratinocyte Serum Free Medium (Invitrogen), which containing 0.05 mg/ml BPE and 5 ng/ml EGF. Cells were cultured at 37 °C with 5% CO2.

### circRNA array

Total RNA from each sample was extracted using TRIzol (Invitrogen, CA, USA) following the instructions of the manufacturer. The concentration and quality of total RNA were evaluated using the NanoDrop ND-1000. Then, total RNAs were digested with RNase R (Epicentre, Inc.) to remove linear RNAs and enrich circular RNAs, followed by circRNAs amplification. The Arraystar Human circRNA Array (8 × 15 K, Arraystar) was used to hybridized circRNAs. The arrays were then scanned by the Agilent Scanner G2505C. Agilent Feature Extraction software (version 11.0.1.1) was used to analyze acquired array images. Quantile normalization and subsequent data processing were performed using the R software limma package.

### m^6^A sequencing

Total RNA from C4-2B cells transfected with control, si-circPDE5A, vector, and circPDE5A plasmids was extracted using TRIzol (Invitrogen, Carlsbad, CA, USA) following the instructions of the manufacturer. The concentration and quality of total RNA were evaluated using the NanoDrop ND-1000. Dynabeads Oligo (dT)25–61,005 (Thermo Fisher, CA, USA) was used to purify poly (A) RNA. Then the poly(A) RNA was fragmented and incubated with an m^6^A-specific antibody. The IP RNA was reverse transcribed to cDNA followed by the synthesis of U-labeled second-stranded DNAs. Adapters were then ligated to the fragments, followed by amplification with PCR. At last, we performed the 2 × 150 bp paired-end sequencing (PE150) on an illumina Novaseq™ 6000 (LC-Bio Technology CO., Ltd., Hangzhou, China) following the recommended protocol of the vendor.

### Plasmid and oligonucleotide transfection

All the plasmids, which include circPDE5A, FOXO4, eIF4A3, WTAP, and EIF3C, used in this study were purchased from Geenchem (Shanghai, China). Lipofectamine 3000 (Invitrogen) were used to transfect plasmids into PCa cells according to the manufacturers’ instruction. circPDE5A specific siRNAs were designed and synthesized by RiboBio (Guangzhou, China). FOXO4, eIF4A3, WTAP, YTHDF1, and EIF3C specific siRNAs were purchased from GenePharma (Shanghai, China). siRNAs transfection was performed by RNAimax (Invitrogen). The circPDE5A knockdown and overexpression lentivirus were also purchased from Geenchem (Shanghai, China). The sequences of siRNAs in this study were listed in the Supplementary Table S3.

### RNA extraction, treatment with RNase R, and quantitative real-time PCR assays

Total RNA from tissues or PCa cell lines was extracted using TRIzol reagent (Invitrogen, CA, USA). Cytoplasmic and nuclear fractions from C4-2B and 22Rv-1 cells were isolated using the Cytoplasmic & Nuclear RNA Purification Kit (Norgen Biotek). Total RNA was incubated with or without 3 U/ug of RNase R (Epicentre, WI, USA) at 37 °C for 5 minutes. First-strand cDNA was synthesized using HiFiScript cDNA Synthesis Kit (CWBio). Real-time quantitative PCR (RT-qPCR) analysis was performed using the SYBR Green method on a LightCycler® 480 System (Roche) according to the manufacturers’ instructions. The relative expression of mRNAs and circRNAs was calculated using 2^–ΔΔ^ Ct method. The specific primers are listed in the Supplementary Table S3.

### Western blot assay

Western blot assay was conducted based on the previous report [21]. Briefly, the proteins were extracted and separated by 10% SDS-PAGE, followed by transfer to the PVDF membrane. The membrane was incubated with primary antibodies overnight at 4 °C. The blots were then incubated with secondary antibodies and detected using the ECL chemiluminescent detection system. The primary antibodies information is listed in the Supplementary Table S3.

### Chromatin immunoprecipitation assay (ChIP)

CHIP assay was performed using the SimpleChIP® Enzymatic Chromatin IP Kit (CST, USA) according to the manufacturers’ instructions. Briefly, C4-2B and 22Rv-1 cells were cross-linked with 1% formaldehyde and quenched with glycine. Then, the cells were lysed and sonicated to the ~ 200–500 bp DNA fragments. FOXO4 antibody and control IgG antibody were used to precipitate the specific DNA fragment. After being washed with four times and decrosslinked, the precipitated DNA was amplified and detected by qRT-PCR. The specific CHIP primers are listed in the Supplementary Table S3.

### Luciferase reporter assay

For the PDE5A promoter-luciferase reporter assay, we constructed the luciferase reporter vectors containing the promoter region of PDE5A (− 2000 bp to TSS) or mutant promotor region of PDE5A without FOXO4-binding sites. C4-2B and 22Rv-1 cells were transfected with luciferase reporter vector, WT, or mutant plasmids plus vector or FOXO4 overexpression plasmids. After 48 h, the luciferase activity was measured using the Dual-Luciferase Reporter Assay System (Promega) according to the manufacturer’s instructions.

### Immunofluorescence

C4-2B and 22Rv-1 cells with circPDE5A knockdown or overexpression were cultured on the cover glasses. The cells were fixed with 4% paraformaldehyde for 15 min and permeabilised with 0.25% Triton-X100 for 10 min. The cells were then blocked with 5% FBS for an hour and then incubated with a specific antibody at 4 °C overnight. Followed by incubating with a corresponding secondary antibody. DAPI was used to mark the nucleus.

### RNA fluorescence in situ hybridization (FISH)

Cy3-labeled circPDE5A probe was designed and synthesized by GenePharma (Shanghai, China). The oligonucleotide sequence was available in Supplementary Table S3. FISH analysis was performed with a FISH kit (GenePharma) according to the manufacturer’s instructions. The signals were measured using the fluorescence microscope (Leica, Wetzlar, Germany).

### Animal model and in vivo imaging

2 × 10^6^ stably transfected 22Rv-1 cells were injected via the tail vein into BALB/c nude mice. After 8 weeks, tumor metastasis loci were measured via in vivo imaging system (IVIS). After 8 weeks, the mice were euthanized, the lungs were surgically dissected and embedded in paraffin. Then, the specimens were stained with hematoxylin and eosin.

### Transwell assay

Briefly, after 48 h transfected with specific oligonucleotides or plasmids, the C4-2B and 22Rv-1cells were suspended with RPMI 1640 medium without FBS. Then, 6 × 10^4^ cells were seeded into the upper chamber, while the lower chamber was plated into a 650 ul medium with 10% FBS. After 24 hours, the migrated cells were fixed and stained crystal violet (0.3%) at room temperature. Finally, the filter membrane was photographed and counted in three random fields.

### Dot blot

A total of 250 ng or 500 ng RNA was denatured at 95 °C for 3 minutes and loaded onto the N+ membranes (GE Health). The RNA and membrane were cross-linked under UV light exposure. The membrane was then washed using PBS containing 0.1% Tween, blocked with 5% milk, and incubated with an m^6^A antibody overnight at 4 °C. Next day, the membrane was incubated with a secondary antibody and detected using the ECL chemiluminescent detection system. 0.02% methylene blue was used as the loading control.

### RNA pulldown assay

circRNA pulldown assay was performed based on publication by Du et al.’s papers [22, 23]. Briefly, Biotin-labeled circRNA probe was synthesized by Tsingke (Beijing, China), and the sequence was complemented to the junction site of the circPDE5A (listed in the Supplementary Table S3). 10^7^ PCa cells were lysed in 500 ul IP buffer, and incubated with the circPDE5A probe for 2 hours at room temperature. Then, 50 ul Streptavidin C1 magnetic beads (Invitrogen) were added to the reaction for another an hour of incubation. Finally, the beads were washed five times, followed by western blot detection.

### RNA immunoprecipitation (RIP)

The RIP assay was performed using Magna RIP Kit (Millipore, USA) according to the manufactures’ guidelines. Briefly, 2 × 10^7^ PCa cells were harvested and lysis in RIP lysis buffer. After centrifugation at 4 °C, the supernatant was incubated with specific antibodies and negative control IgG at room temperature. The beads-antibody complex was then washed and incubated with Proteinase K. The immunoprecipitated RNA was purified and detected by RT-qPCR.

### Polysome profiling

C4-2B and 22Rv-1 cells were treated with 100 μg/ml cycloheximide for 15 minutes and then lysed with lysis buffer. The lysate was collected and ultracentrifuged at 4 °C for 4 hours with a 10–50% sucrose gradient solution. The samples were collected with the Gradient Station. And each fractionation RNA was extracted with TRIzol reagent and subjected to the RT-qPCR assay.

### Statistical analysis

The results were presented as the means ± SD. All the differences were calculated using SPSS by SPSS version 18.0 (Chicago, USA). The figures were plotted using GraphPad Prism 7 (GraphPad Software, Inc., CA). The Wilcoxon matched-pairs signed-rank test was used to estimate the differential expression in clinical samples. The student’s t test or the unpaired two sides t test was used to evaluate the differences between different groups. The *p* value < 0.05 was considered to be statistically significant.

## Results

### Identification and characterization of circPDE5A

**Fig. 1:**
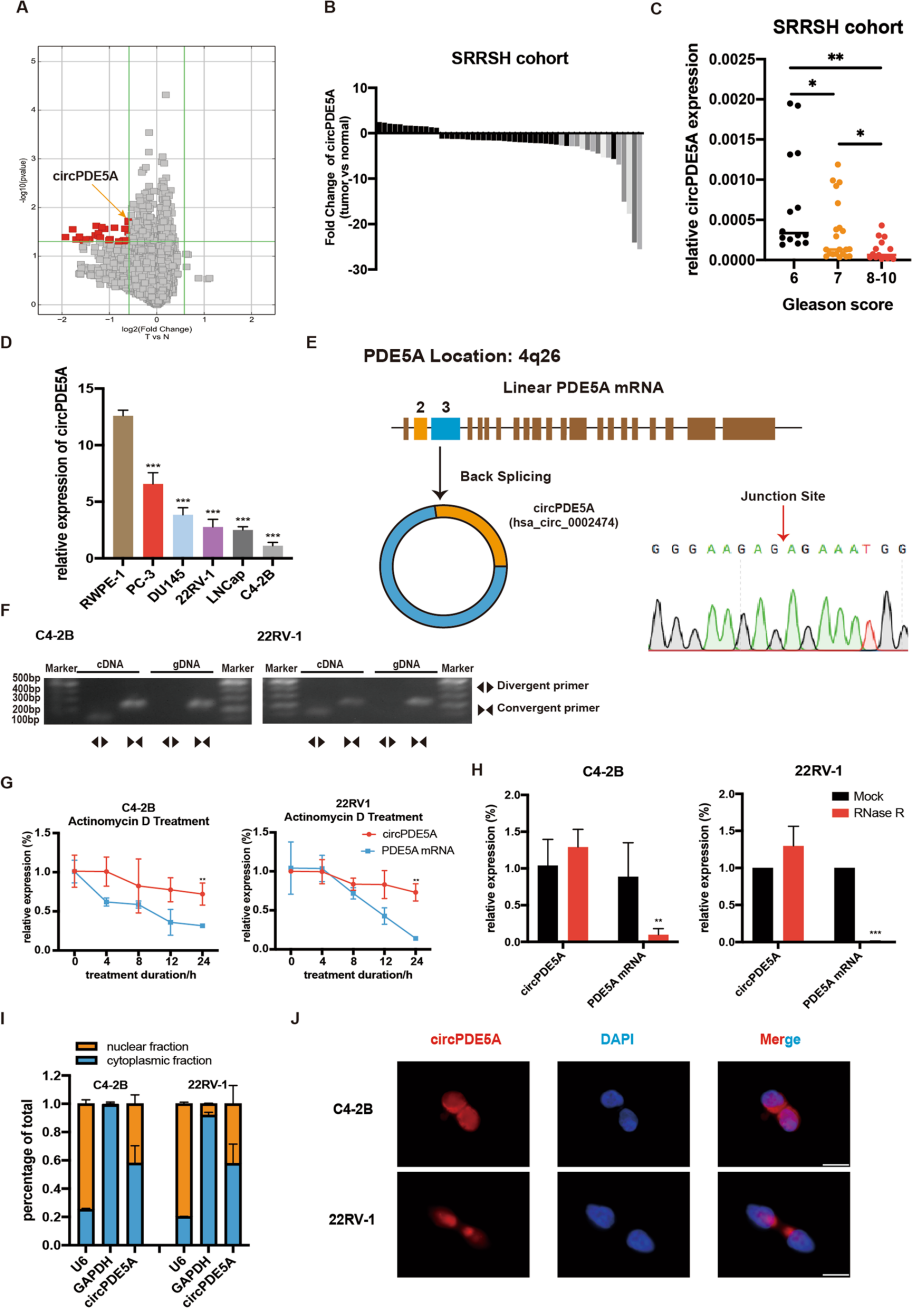
Identification and characterization of circPDE5A. **A,** Volcano plot showing differentially expressed circRNAs between five paired prostate cancer tissues and paired adjacent normal tissues. **B,** Relative expression levels of circPDE5A in 50 paired prostate cancer tissues and adjacent normal tissues. **C,** Relative expression levels of circPDE5A in 50 patients having different Gleason scores (6, 7, and 8–10). **D,** Relative expression levels of circPDE5A in prostate cancer cell lines and normal prostate epithelial cell lines. **E,** Genomic location of circPDE5A. circPDE5A was formed following back-splicing of exon 19 and 20 of PDE5A and the junction site was verified using sanger sequencing. **F,** PCR of gDNA and cDNA using divergent primer and convergent primers. **G,** Relative expression levels of circPDE5A and PDE5A mRNA after actinomycin D treatment for 0 h, 4 h, 8 h, 12 h, and 24 h. **H,** Relative expression levels of circPDE5A and PDE5A mRNA after RNase R treatment. **I,** Relative expression levels of circPDE5A in nuclear and cytoplasmic fractions. **J,** The cellular distribution of circPDE5A using fluorescence in situ hybridization. Scale bars, 5 μm. Data represents mean ± S.D. from three independent experiments. *, *p* < 0.05; **, *p* < 0.01

To investigate the potential role of circRNA in prostate cancer, we firstly performed a circRNA array in five paired samples of PCa by Arraystar Human circRNA array (Supplementary Table S1). A total of 27 dysregulated circRNAs were identified in PCa tissues (Fig. 1A). Among these circRNAs, 19 circRNAs could be found in the circbase database. However, only 8 of 19 circRNAs could be amplified in cDNA using the specific primers. Next, we examined the expression of these circRNAs in 15 paired prostate cancer and adjacent normal tissues. The results showed that only hsa_circ_0002474, derived from PDE5A, designated as circPDE5A, was downregulated in PCa tissues compared to adjacent normal tissues (Supplementary Fig. 1A). We then confirmed the expression of circPDE5A in 50 paired samples of PCa by qRT-PCR. The results demonstrated that hsa_circ_0002474 was significantly downregulated in prostate cancer compared with adjacent normal tissues (Fig. 1B). As shown in Fig. 1C, the expression of circPDE5A in patients with PCa was negatively correlated with Gleason score. Moreover, circPDE5A was downregulated in PCa cell lines compared with normal prostate epithelial cell line (Fig. 1D). These results reveal that the differential expression of circPDE5A might play a role in PCa progression.

circPDE5A is a 679-nt circRNA generated from the exon 2 to 3 of the PDE5A gene, and the junction site was confirmed using sanger sequencing (Fig. 1E). RT-PCR analysis showed that the divergent primer could amplify circPDE5A in cDNA reverse transcribed by total RNA, but not genome DNA (Fig. 1F). After treatment of actinomycin D, circPDE5A demonstrated more stability compared to PDE5A mRNA (Fig. 1G). RNase R was utilized to detect the stability of circPDE5A to examine the circular characterization of circPDE5A further. The results revealed that circPDE5A exhibited high resistance to RNase R digestion, whereas the liner RNA of PDE5A mRNA was mostly degraded (Fig. 1H). Next, both nuclear and cytoplasmic fractionation and FISH assay demonstrated that circPDE5A was localized in both the cytoplasm and nucleus (Fig. 1I, J). These results suggest the circularity and localization of circPDE5A in PCa cells.

### circPDE5A restrains prostate cancer cells metastasis both in vivo and in vitro

**Fig. 2:**
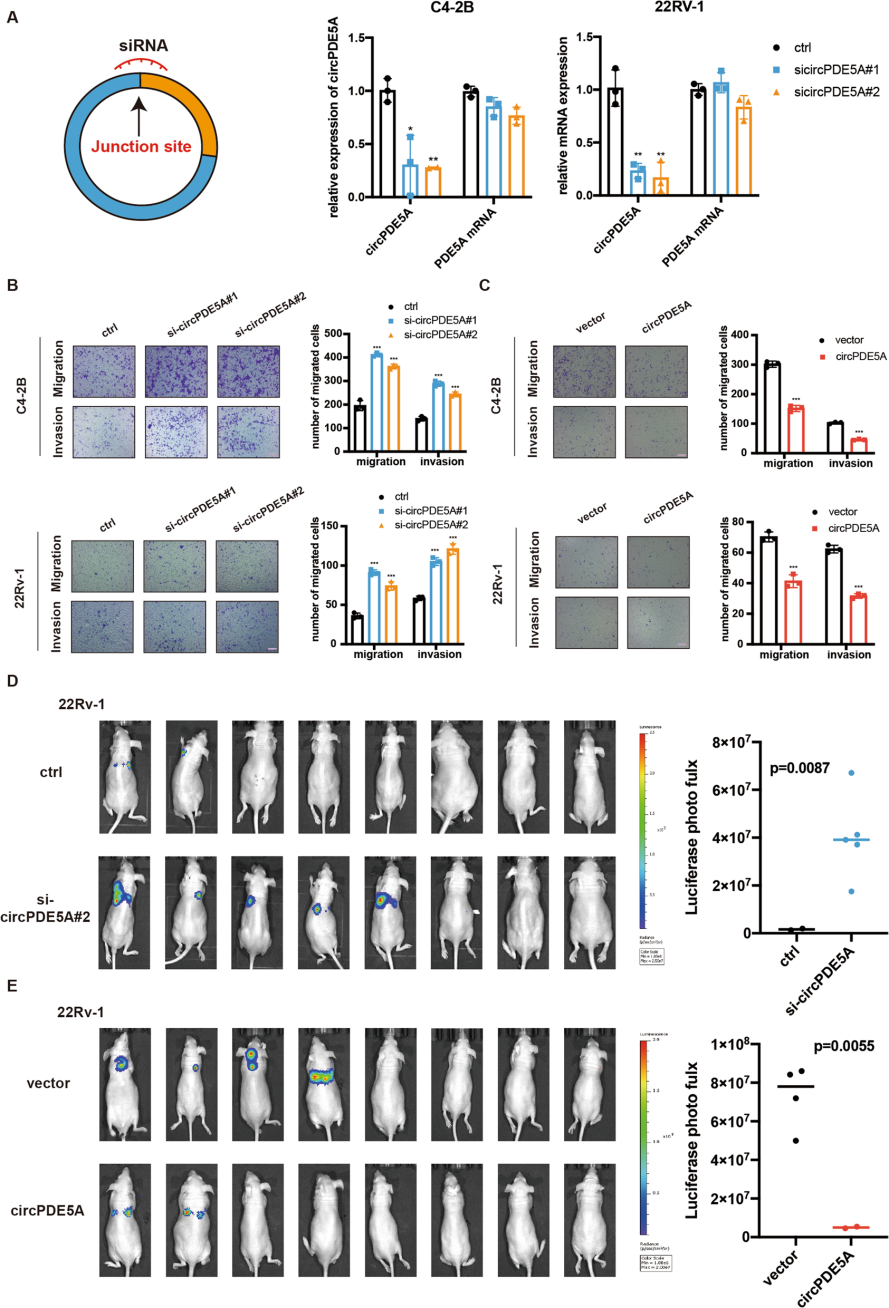
circPDE5A restrains prostate cancer cell metastasis both in vivo and in vitro. **A,** The knockdown efficiency of circPDE5A siRNAs in C4-2B and 22Rv-1 cells. **B,** Transwell assay in C4-2B and 22Rv-1 cells transfected with circPDE5A siRNAs or the siRNA control. Scale bars, 5 μm. **C,** Transwell assays in C4-2B and 22Rv-1 cells transfected with circPDE5A overexpression plasmids or vector plasmids. Scale bars, 5 μm. **D,** Left: the metastatic ability of stably knocking down circPDE5A and control 22Rv-1 cells using the nude mice tail vein metastasis model; right: quantification of the radiance intensity of understudied cells. **E,** Left: the metastatic ability of stably overexpressing circPDE5A and control 22Rv-1 cells using nude mice tail vein metastasis model; right: the quantification of radiance intensity of understudied cells. Data represents mean ± S.D. from three independent experiments. *, *p* < 0.05; **, *p* < 0.01; ***, *p* < 0.001

To investigate the role of circPDE5A in PCa progression, we designed specific small-interfering RNAs (siRNAs) which targeting back-splicing junction sites of circPDE5A. qRT-PCR analysis showed that circPDE5A siRNAs specifically reduced the circPDE5A expression, while having little effect on PDE5A mRNA expression (Fig. 2A). Meanwhile, we stably overexpressed circPDE5A in C4-2B and 22Rv-1 cell lines using lentivirus plasmids, and the overexpression efficiency was evaluated (Supplementary Fig. 2A). Next, we investigated whether circPDE5A influenced PCa cells proliferation. The CCK-8 assay revealed that neither downregulation nor overexpression of circPDE5A had little effect on PCa cells proliferation (Supplementary Fig. 2B, C). Then, a transwell assay was performed to explore the ability of circPDE5A on migration and invasion. The results suggested that circPDE5A knockdown significantly promoted migration and invasion of PCa cells (Fig. 2B), while circPDE5A overexpression evidently inhibited migration and invasion of PCa cells (Fig. 2C). These results demonstrate that circPDE5A restrains PCa cells metastasis.

To further confirm the role of circPDE5A in vivo, xenograft tumor animal assay was performed using 22Rv-1 cells stably transfected with circPDE5A or vector. The result showed that circPDE5A overexpression has little effect on prostate cancer cells proliferation in vivo (Supplementary Fig. 2D), consistent with the in vitro assay. Next, we utilized a nude mice tail vein metastasis model to investigate the role of circPDE5A on metastasis of PCa cells in vivo. circPDE5A stably knockdown or overexpression 22Rv-1 cells were injected into the tail vein of nude mice. We found that 22Rv-1 cells with circPDE5A knockdown formed metastasis foci in 62.5% (5/8) mice in a period of 6 weeks after injection, while 22Rv-1 control cells only formed metastasis foci in 25% (2/8) mice (Fig. 2D). However, 22Rv-1 cells with circPDE5A overexpression formed fewer metastasis foci (2/8) than the control group (4/8) (Fig. 2E). Overall, these findings reveal that circPDE5A restrains metastasis of PCa cells in vivo.

### circPDE5A binds to WTAP and regulates its m^6^A methylation activity

**Fig. 3:**
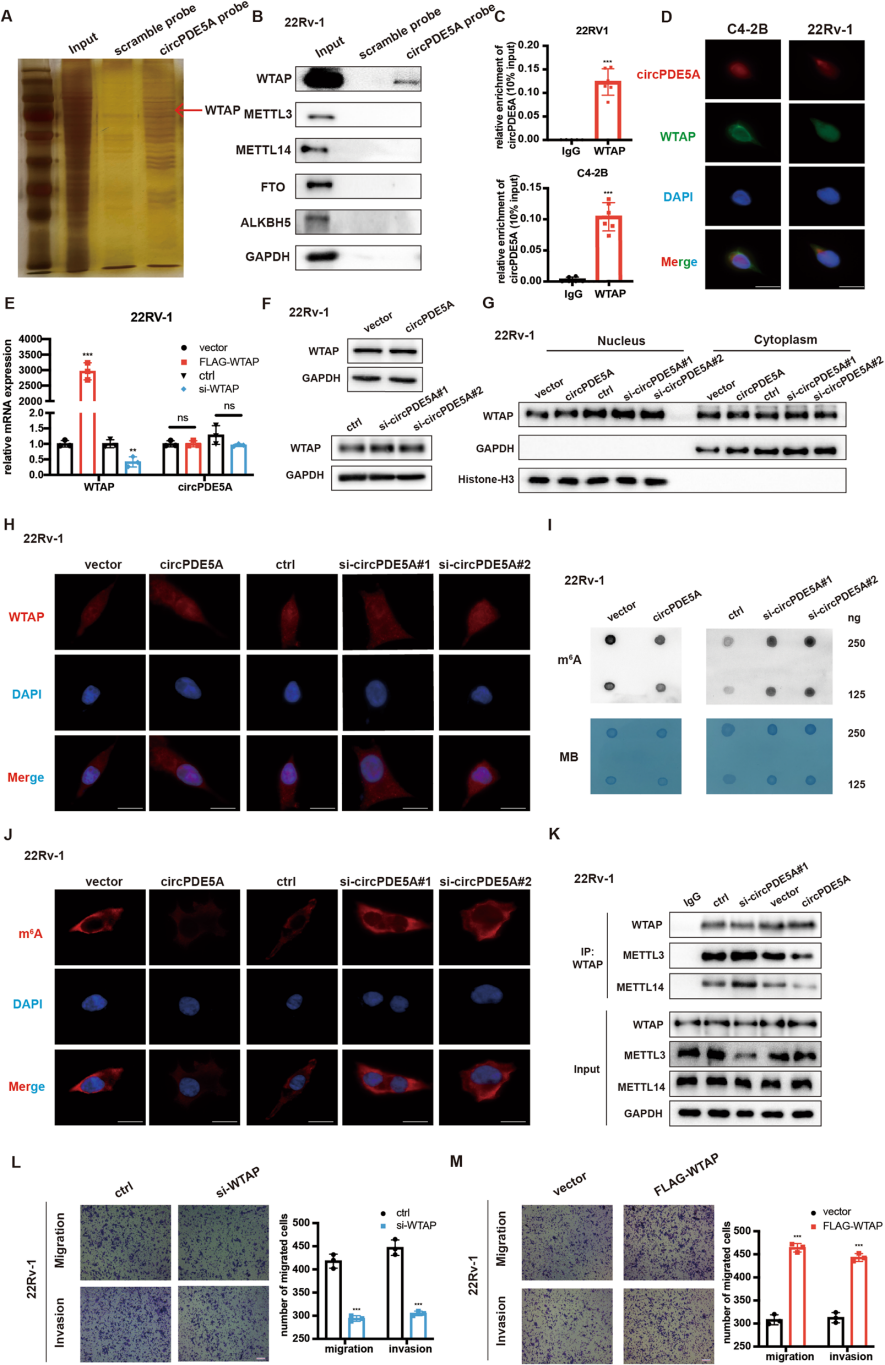
circPDE5A binds to WTAP and regulates its m6A methylation activity. **A,** Identification of circPDE5A binding proteins using silver staining. **B,** Detection of the interaction between circPDE5A and m^6^A methyltransferases or demethylases through western blotting. **C,** The binding capacity between circPDE5A and WTAP in C4-2B and 22Rv-1 cells through RNA-IP assay. **D,** IF and FISH assays showing the colocalization of WTAP and circPDE5A in C4-2B and 22Rv-1 cells. Scale bars, 5 μm. **E,** Analysis of the expression of circPDE5A in 22Rv-1 cells with WTAP overexpression or knockdown through RT-qPCR. **F,** Analysis of the expression of WTAP in 22Rv-1 cells with circPDE5A overexpression or knockdown by western blot. **G,** Analysis of the cellular distribution of WTAP in 22Rv-1 cells with circPDE5A overexpression or knockdown by western blot. **H,** Analysis of the cellular distribution of WTAP in 22Rv-1 cells with circPDE5A overexpression or knockdown through immunofluorescence. Scale bars, 10 μm. **I,** Dot blot showing the total m^6^A modification level in 22Rv-1 cells with circPDE5A overexpression or knockdown. **J,** Immunofluorescence showing the total m^6^A modification level in 22Rv-1 cells with circPDE5A overexpression or knockdown. Scale bars, 10 μm. **K,** Co-IP assay showing the binding capacity between WTAP and METTL3 or METTL14 in 22Rv-1 cells after circPDE5A overexpression or knockdown. **L, M,** Transwell assay showing the migration and invasion ability with cells subjected to WTAP knockdown (**L**) or overexpression (**M**) in 22Rv-1 cells. Scale bars, 5 μm. Data represents mean ± S.D. from three independent experiments. ***, *p* < 0.001

We next explored the detailed mechanism of circPDE5A in PCa metastasis. Since many circRNAs were reported to function by serving as “miRNA sponges”, we performed AGO2-RIP assay to examined whether circPDE5A could sponge miRNAs. However, the results showed that circPDE5A could not bind to AGO2 protein (Supplementary Fig. 3A), indicating that circPDE5A may not function as “miRNA sponges”. Next, we hypothesized whether circPDE5A exerted its roles by binding to the functional proteins. RNA-pulldown assay followed by qRT-PCR analysis revealed that circPDE5A probe could specifically bind to circPDE5A (Supplementary Fig. 3B). Then, the protein extraction in the RNA pulldown assay was separated by SDS-PAGE, and followed by silver staining (Fig. 3A), demonstrating that circPDE5A could specifically bind to many proteins. Moreover, RNA pulldown assay followed by mass spectrometry was performed to detect the circPDE5A binding proteins, and a total of 123 proteins were identified. Among these proteins, WTAP attracted our attention. WTAP was a key component of the m^6^A methyltransferase. Since the function of circRNAs in m^6^A modification remains elusive [24]. We, therefore examined the role of circPDE5A/WTAP complex in prostate cancer. We observed that circPDE5A bind directly to WTAP, but not to other m^6^A methyltransferases and demethylases METTL3, METTL14, FTO, and ALKBH5 (Fig. 3B). The RIP-qPCR assay was then used to confirmed the binding of circPDE5A and WTAP (Fig. 3C). Moreover, FISH and immunofluorescence assay was performed to confirm that circPDE5A was colocalized with WTAP protein in cells (Fig. 3D). Based on the interaction between circPDE5A and WTAP, we tested whether WTAP affect circPDE5A expression. However, the qRT-PCR analysis revealed that WTAP had little effect on circPDE5A expression (Fig. 3E and Supplementary Fig. 3C). Furthermore, changing circPDE5A expression did not alter the WTAP expression and localization (Fig. 3F–H, Supplementary Fig. 3D–F). Since WTAP was a well-known N6-methyadenosine methyltransferase which promoted m^6^A modification by recruiting METTL3 and METTL14 [25]. We speculated that the interaction between circPDE5A and WTAP could affect WTAP m^6^A activity. Therefore, we detected m^6^A levels in circPDE5A overexpression or knockdown PCa cells. The dot blot assay demonstrated that circPDE5A overexpression decreased the global m^6^A level, while circPDE5A knockdown increased the global m^6^A level in PCa cells (Fig. 3I and Supplementary Fig. 3G). The m^6^A immunofluorescence assay also received the same results (Fig. 3J and Supplementary Fig. 3H). Next, we explored how circPDE5A interfered the WTAP-dependent m^6^A modification in PCa. The CO-IP assay results showed that circPDE5A overexpression decreased the interaction between METTL3 and METTL14, while silencing of circPDE5A increased the interaction between METTL3 and METTL14 (Fig. 3K), suggesting that circPDE5A may block WTAP m^6^A activity via interfering the formation of METTL3-METTL14-WTAP complex. Meanwhile, the transwell assay revealed that silencing of WTAP inhibited PCa migration and invasion, at the same time, overexpression of WTAP promoted PCa migration and invasion (Fig. 3L, M, Supplementary Fig. 3I, 3 J), which was consistent with our previous findings. These results reveal that circPDE5A inhibits PCa metastasis via interacting with WTAP and blocks its m^6^A activity.

### circPDE5A inhibits WTAP-mediated m^6^A modification of EIF3C mRNA and restrains its translation

**Fig. 4:**
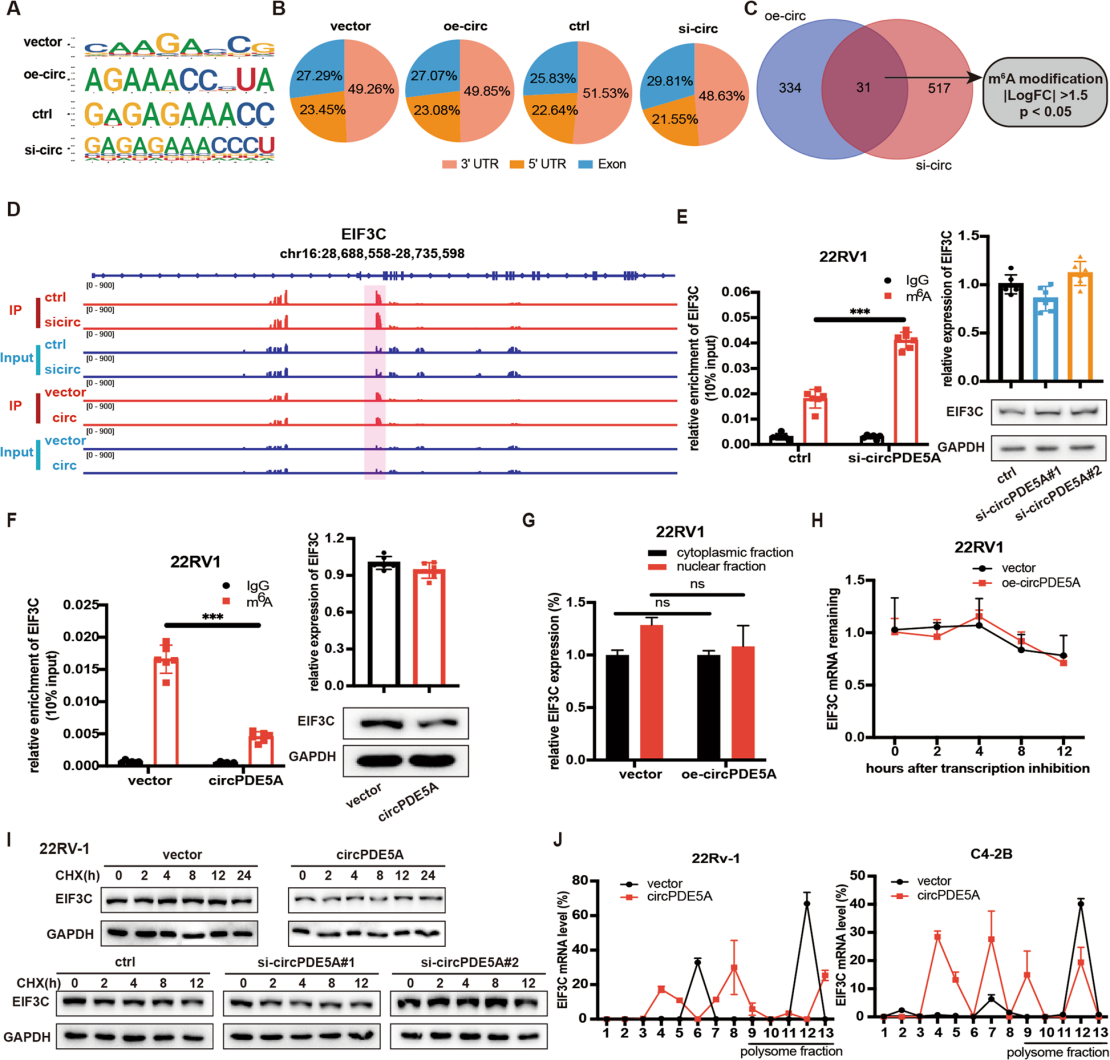
circPDE5A decreases the m6A modification of EIF3C mRNA and restrains its translation. **A,** m^6^A motifs identified using MeRIP-seq. **B,** The distribution of m^6^A modification in 3′UTR, 5′UTR, and exon regions after circPDE5A overexpression or knockdown. **C,** Venn diagram showing the overlapped altered m^6^A modification genes after circPDE5A overexpression or knockdown. **D,** Integrative genomics viewer displaying the results of EIF3C m^6^A modification distribution in MeRIP-seq. **E, F,** Left: MeRIP assay showing the m^6^A modification level of EIF3C in 22Rv-1 cells with circPDE5A knockdown (**E**) or overexpression (**F**); right: the mRNA and protein expression of EIF3C in 22Rv-1 cells with circPDE5A knockdown (**E**) or overexpression (**F**). **G,** RT-qPCR assay showing the cellular distribution of EIF3C in 22Rv-1 cells with circPDE5A overexpression. **H,** RT-qPCR assay showing the EIF3C mRNA stability in 22Rv-1 cells with circPDE5A overexpression. **I,** WB assay showing the EIF3C protein stability in 22Rv-1 cells with circPDE5A overexpression or knockdown. **J,** RT-qPCR assay showing the relative level of EIF3C mRNA in gradient fractions with circPDE5A overexpression in C4-2B and 22Rv-1 cells. Data represents mean ± S.D. from three independent experiments. ***, *p* < 0.001

To figure out the molecular mechanism in the regulatory effect of circPDE5A on m^6^A modification. We performed m^6^A methylated RNA immunoprecipitation sequencing (MeRIP-seq) in vector, circPDE5A overexpression, control and circPDE5A knockdown groups (Fig. 4A, B). To narrow down the scope of downstream genes, we integrated the circPDE5A overexpression and circPDE5A knockdown data (criteria: m^6^A peak |log_2_FC| > 1.5). A total of 31 m^6^A peak dysregulated genes is shown in the Venn diagram (Fig. 4C). Among these genes, 16 mRNAs were selected as the candidate downstream targets that might participated in the cancer progression. Then, we performed MeRIP-qPCR to evaluate the effect of circPDE5A on these candidate targets. The results showed that only the m^6^A modification of EIF3C was upregulated after circPDE5A knockdown and downregulated after circPDE5A overexpression (Supplementary Fig. 4A). These data suggested that EIF3C was the potential target of circPDE5A/WTAP complex (Fig. 4D). Next, we explored whether circPDE5A regulated EIF3C expression. The m^6^A modification and protein expression of EIF3C were elevated, while the EIF3C mRNA level has little change in circPDE5A knockdown PCa cells compared to scramble cells (Fig. 4E and Supplementary Fig. 4B). By contrast, the m^6^A modification and protein expression of EIF3C were decreased, while the EIF3C mRNA level had little change in circPDE5A overexpression PCa cells compared to vector cells (Fig. 4F and Supplementary Fig. 4C). Next, we explored why circPDE5A altered the EIF3C protein level but not the mRNA level. Since it is reported that the m^6^A modification of mRNA could change its cellular distribution [26], we conducted nuclear and cytoplasmic fractionation, and the results suggested that circPDE5A overexpression did not alter the EIF3C mRNA cellular distribution (Fig. 4G and Supplementary Fig. 4D). Similarly, overexpression or knockdown of circPDE5A did not change the EIF3C mRNA stability (Fig. 4H and Supplementary Fig. 4E, F). Then, protein translation inhibitor cycloheximide (CHX) was added in circPDE5A knockdown or overexpression PCa cells. The results revealed that neither knockdown nor overexpression of circPDE5A had little effect on EIF3C protein stability, excluding the possibility that circPDE5A affects EIF3C protein stability (Fig. 4I and Supplementary Fig. 4G). Finally, polysome profiling demonstrated that overexpression of circPDE5A considerably decreased the enrichment of EIF3C mRNA in the polysome fractions but increased in the non-polysome fractions (Fig. 4J), while knockdown of circPDE5A increased the enrichment of EIF3C mRNA in the polysome fractions (Supplementary Fig. 4H), supporting that circPDE5A regulated EIF3C protein expression via interfering with its translation.

**Fig. 5:**
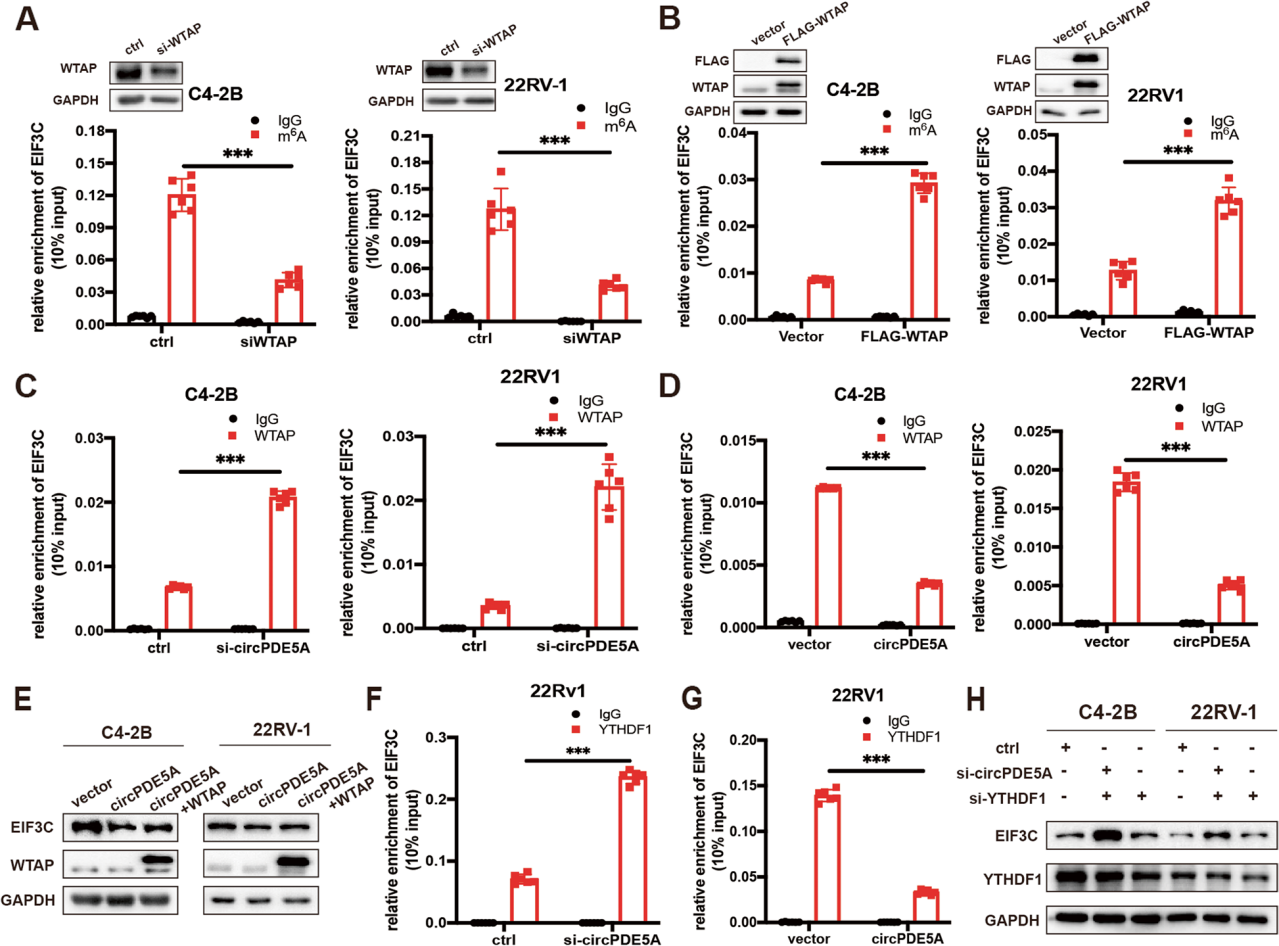
circPDE5A regulates the m6A modification of EIF3C mRNA thorough WTAP. **A,** MeRIP assay showing the m^6^A modification level of EIF3C with WTAP knockdown in C4-2B and 22Rv-1 cells. **B,** MeRIP assay showing the m^6^A modification level of EIF3C with WTAP overexpression in C4-2B and 22Rv-1 cells. **C,** WTAP-RIP assay showing the binding capacity between EIF3C mRNA and WTAP with circPDE5A knockdown in C4-2B and 22Rv-1 cells. **D,** WTAP-RIP assay showing the binding capacity between EIF3C mRNA and WTAP with circPDE5A overexpression in C4-2B and 22Rv-1 cells. **E,** Western blotting assay showing the protein levels of EIF3C and WTAP in circPDE5A overexpression C4-2B and 22Rv-1 cells with the overexpression of WTAP. **F, G,** YTHDF1-RIP assay showing the binding capacity between YTHDF1 and EIF3C mRNA in 22Rv-1 cells with circPDE5A knockdown (**F**) or overexpression (**G**). **H,** Western blotting assay showing the EIF3C and YTHDF1 protein expression in circPDE5A knockdown C4-2B and 22Rv-1 cells with the silencing of YTHDF1. Data represents mean ± S.D. from three independent experiments. *, *p* < 0.05; **, *p* < 0.01; ***, *p* < 0.001

Given that circPDE5A regulated EIF3C translation via altering its m6A modification, we first verified whether WTAP played an indispensable role in this process. MeRIP-qPCR assay suggested that silencing WTAP decreased the EIF3C m^6^A modification significantly, while overexpression of WTAP increased the EIF3C m^6^A modification in PCa cells (Fig. 5A, B). Next, WTAP-RIP-qPCR analysis showed that silencing circPDE5A enhanced the binding capacity between WTAP and EIF3C mRNA (Fig. 5C). However, circPDE5A overexpression impaired this interaction (Fig. 5D). Meanwhile, we found that WTAP overexpression could reverse circPDE5A induced EIF3C downregulation in protein levels rather than mRNA levels (Fig. 5E and Supplementary Fig. 5A). These results suggested that circPDE5A regulated EIF3C m^6^A modification via interfering with the interaction between WTAP and EIF3C mRNA.

Previous studies had reported that YTHDF1 and IGF2BP1, two m^6^A readers, could regulated protein translation via recognizing the m^6^A modification of mRNA [27, 28]. Thus, western blotting assay was used to explored which reader influenced the expression of EIF3C. The result showed that silencing of YTHDF1, but not IGF2BP1, decreased the protein level of EIF3C significantly (Supplementary Fig. 5B, C), revealing that YTHDF1 might a key reader regulating EIF3C mRNA translation. The YTHDF1-RIP-qPCR assay was then used to prove that YTHDF1 could bind more EIF3C mRNA compared to the IgG control group (Fig. 5F). Meanwhile, the binding of EIF3C and YTHDF1 also was confirmed by the CLIP-seq data (http://starbase.sysu.edu.cn/index.php) (Supplementary Fig. 5D). In addition, the YTHDF1-RIP-qPCR assay showed that silencing of circPDE5A enhanced the binding capacity between YTHDF1 and EIF3C (Fig. 5F and Supplementary Fig. 5E). At the same time, circPDE5A overexpression decreased the YTHDF1 and EIF3C binding capacity (Fig. 5G and Supplementary Fig. 5F), suggesting that the binding capacity between YTHDF1 and EIF3C mRNA depending on the m^6^A modification level of EIF3C mRNA. Also, WB analysis demonstrated that silencing of YTHDF1 abrogated the promoting effect of circPDE5A knockdown in the EIF3C protein level (Fig. 5H). These data demonstrate that circPDE5A regulated EIF3C protein expression in a YTHDF1-dependent manner.

### EIF3C promotes prostate cancer cells metastasis through MAPK pathway

**Fig. 6:**
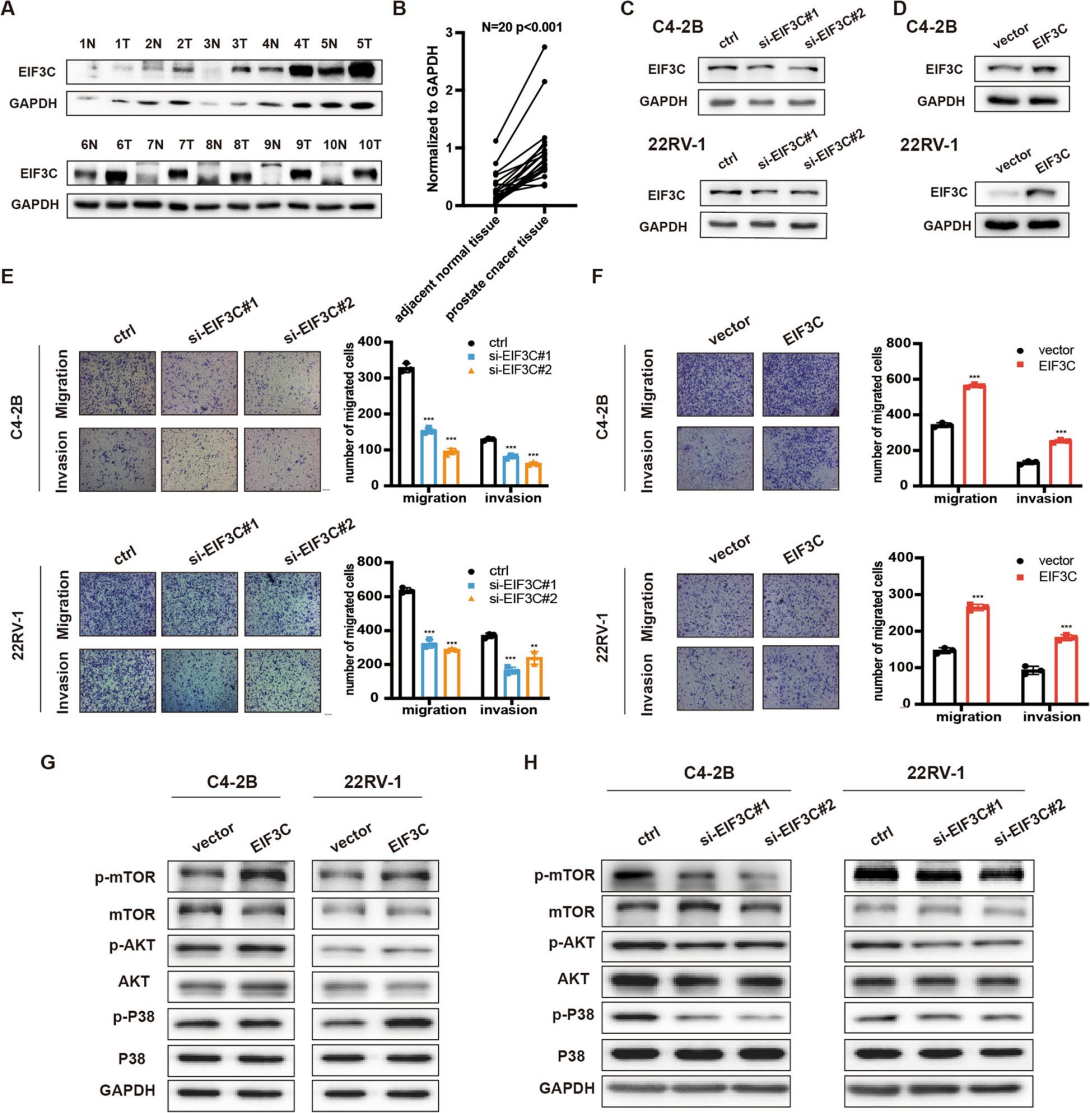
EIF3C promotes prostate cancer cells metastasis through the MAPK pathway. **A,** The relative mRNA expression level of EIF3C in PCa and normal specimens obtained from the TCGA database. **B,** RT-qPCR showing the relative mRNA expression level of EIF3C in our PCa cohort. **C,** Western blotting assay showing the protein expression level of EIF3C in 15 paired PCa and normal specimen. **D,** Quantification of the band intensities during Western blot. **E,** Transwell assay in C4-2B and 22Rv-1 cells transfected with EIF3C siRNAs or the siRNA control. Scale bars, 5 μm. **F,** Transwell assay in C4-2B and 22Rv-1 cells transfected with EIF3C overexpression plasmids or vector. Scale bars, 5 μm. **G, H,** Western blotting showing the effect of EIF3C overexpression (**G**) or knockdown (**H**) on the MAPK pathway proteins in C4-2B and 22Rv-1 cells. Data represents mean ± S.D. from three independent experiments. ***, *p* < 0.001

EIF3C was one of the subunits of EIF3, playing vital roles in translation initiation [29]. Previous studies had revealed that EIF3C was inevitable for tumor progression in many cancer types, including lung cancer, glioma, and ovarian cancer [30–32]. However, the specific role of EIF3C in prostate cancer is still elusive. We found that the protein level EIF3C was upregulated in PCa tissues compared to adjacent normal tissues (Fig. 6A, B). To further investigated the role of EIF3C in PCa progression, we first manipulated the expression of EIF3C in C4-2B and 22Rv-1 cells, and the knockdown or overexpression efficiency was confirmed (Fig. 6C, D, Supplementary Fig. 6A, B). The transwell assay in C4-2B and 22Rv-1 cells demonstrated that silencing of EIF3C significantly restrained the migration and invasion ability of PCa cells (Fig. 6E). In contrast, overexpression of EIF3C promoted the ability of migration and invasion of PCa cells (Fig. 6F). Previous studies revealed that EIF3C promoted cancer progression via regulating MAPK pathway [33–35]. We, therefore, examined the p-mTOR, p-AKT, and p-P38 levels in PCa cells after EIF3C knockdown or overexpression. The WB analysis demonstrated that overexpression of EIF3C elevated the phosphorylation level of mTOR, AKT, and P38 in C4-2B and 22Rv-1 cells (Fig. 6G). However, the phosphorylation level of mTOR, AKT and P38 in PCa cells was decreased after EIF3C knockdown (Fig. 6H). These results reveal that EIF3C participates in PCa progression via the MAPK pathway.

### circPDE5A restrains prostate cancer metastasis via EIF3C

**Fig. 7:**
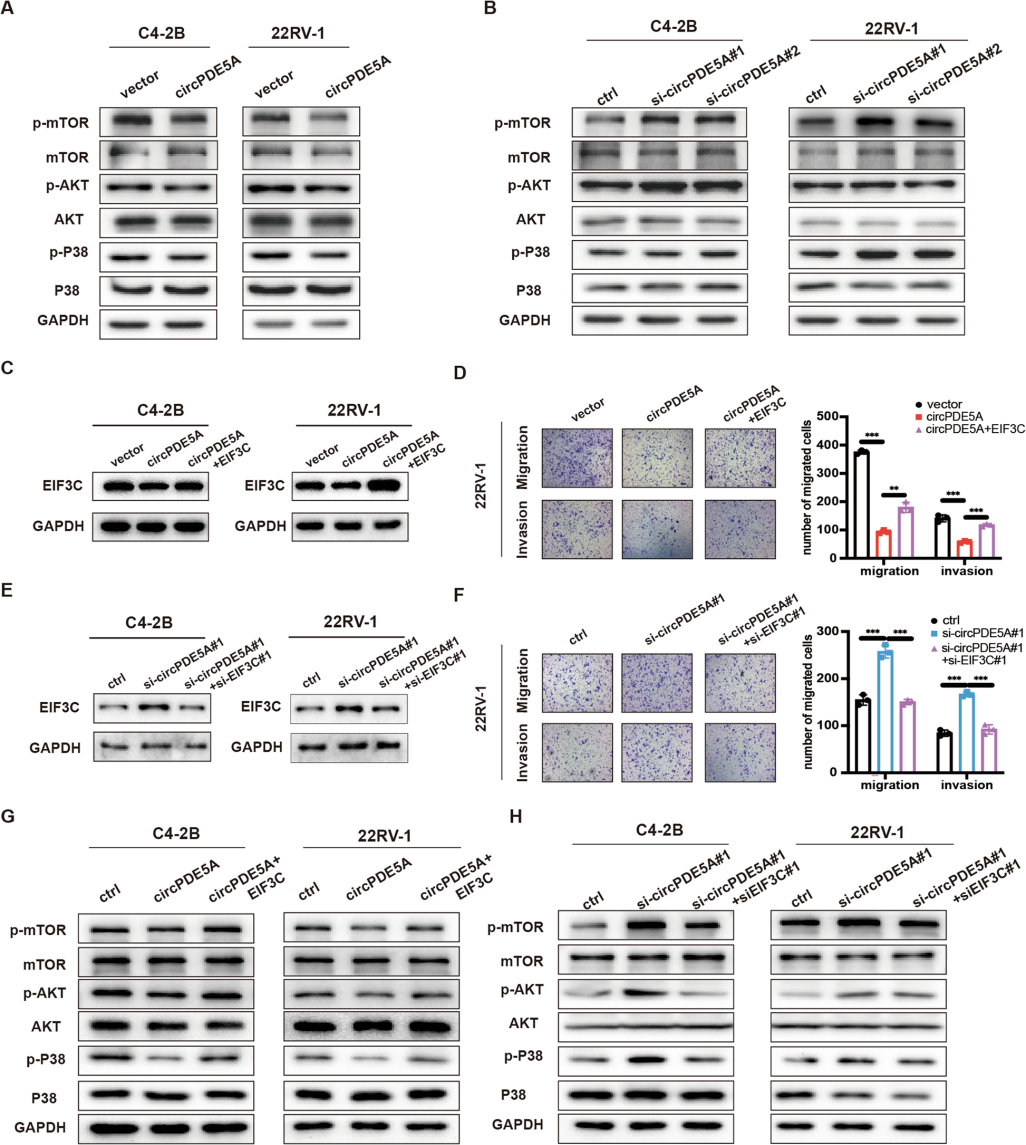
circPDE5A restrains prostate cancer metastasis via EIF3C. **A, B,** Western blotting showing the effect of circPDE5A overexpression (**A**) or knockdown (**B**) on the MAPK pathway proteins. **C,** Western blotting showing the EIF3C protein expression in circPDE5A overexpression C4-2B or 22Rv-1 cells with the overexpression of EIF3C. **D,** Transwell assay showing the migration and invasion ability of 22Rv-1 cells described in (C). Scale bars, 5 μm. **E,** Western blotting showing the EIF3C protein expression in circPDE5A knockdown C4-2B or 22Rv-1 cells with the silencing of EIF3C. **F,** Transwell assay showing the migration and invasion ability of 22Rv-1 cells described in (E). Scale bars, 5 μm. **G,** Western blotting assay showing the effect of EIF3C overexpression on the MAPK pathway proteins in C4-2B or 22Rv-1 cells described in (C). **H,** Western blotting assay showing the effect of EIF3C knockdown on the MAPK pathway proteins in C4-2B or 22Rv-1 cells described in (F). Data represents mean ± S.D. from three independent experiments. **, *p* < 0.01; ***, *p* < 0.001

Since circPDE5A regulated EIF3C protein levels in PCa tissues, we first examined whether circPDE5A also regulated the MAPK pathway. The WB analysis showed that overexpression of circPDE5A decreased the p-mTOR, p-AKT and p-P38 levels in PCa cells while silencing circPDE5A elevated the p-mTOR, p-AKT, and p-P38 levels in PCa cells (Fig. 7A, B). We then overexpressed the EIF3C level in circPDE5A overexpression PCa cells, and the WB analysis showed that the EIF3C expression was restored in PCa cells (Fig. 7C). The transwell assay revealed that circPDE5A overexpression inhibited migration and invasion of PCa cells, while this inhibition effect could be reversed via overexpression of EIF3C (Fig. 7D and Supplementary Fig. 6C). Similarly, silencing EIF3C could rescue the promoting effect when circPDE5A knockdown in PCa cells (Fig. 7F and Supplementary Fig. 6D). The WB analysis also demonstrated that the inhibitory effect of circPDE5A on the MAPK pathway could be reversed by elevating the EIF3C expression in PCa cells (Fig. 7G) and the elevated level of MAPK pathway markers caused by circPDE5A knockdown could be restored by silencing of EIF3C (Fig. 7H). These data reveal that EIF3C is a critical downstream target of circPDE5A in PCa.

### FOXO4 regulates circPDE5A expression in prostate cancer

**Fig. 8:**
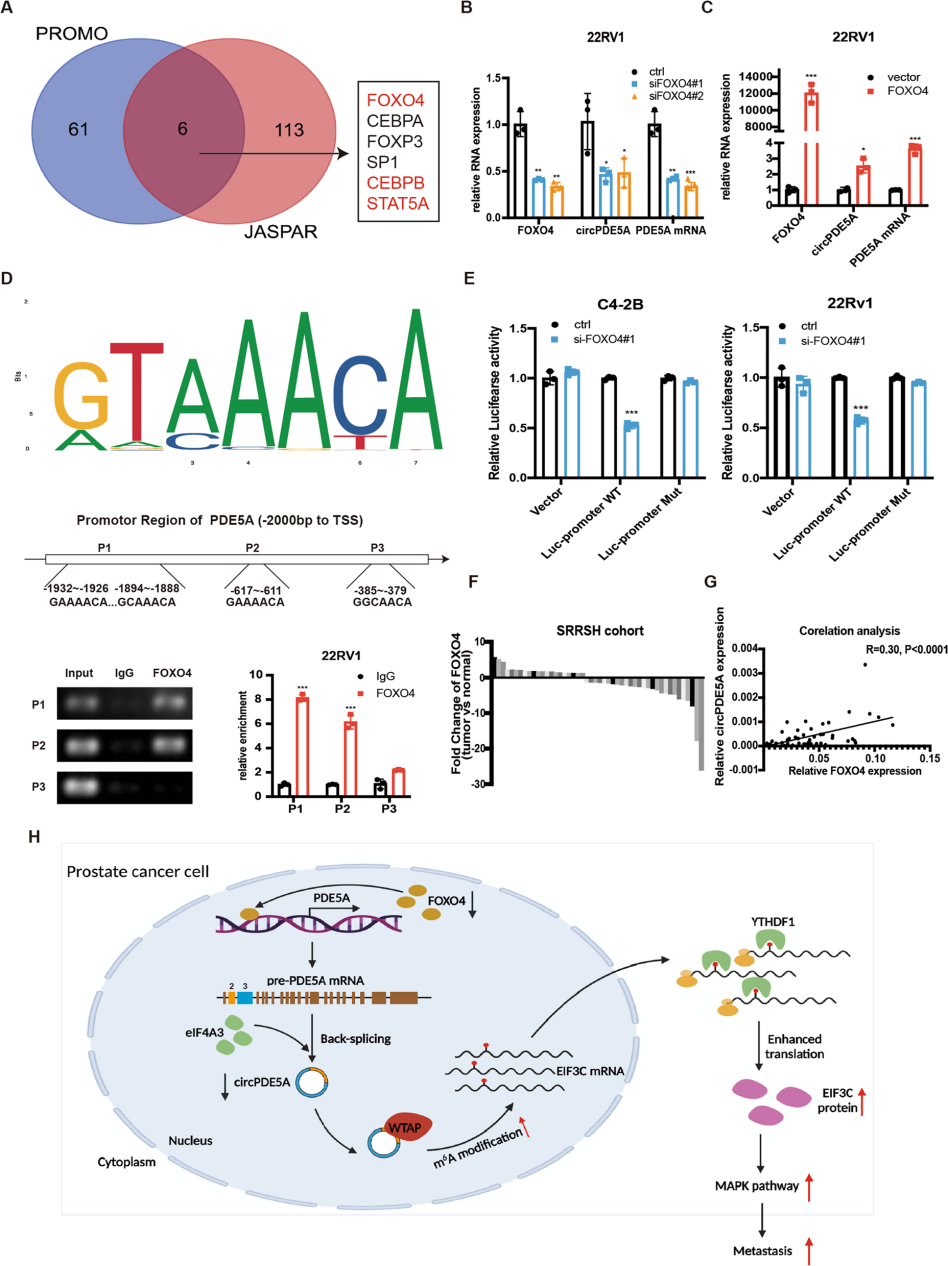
FOXO4 regulate circPDE5A expression in prostate cancer cells. **A,** Venn diagram showing transcriptional factors predicted using JASPAR and PROMO databases. **B,** The expression of circPDE5A and PDE5A mRNA in FOXO4 knockdown 22Rv-1 cells. **C,** The expression of circPDE5A and PDE5A mRNA in FOXO4 overexpression 22Rv-1 cells. **D,** Top: the putative FOXO4 binding motif in PDE5A’s promoter region predicted using the JASPAR database; bottom: the relative enrichment of FOXO4 in three potential binding sites compared with IgG. **E,** The effect of FOXO4 on the luciferase activity of PDE5A’s promoter region using a dual-luciferase reporter assay. **F,** Relative expression levels of FOXO4 in 50 paired prostate cancer tissues and adjacent normal tissues. **G,** Correlation analysis of the expression between FOXO4 and circPDE5A in PCa specimen. **H,** The schematic diagram illustrating the role of circPDE5A in prostate cancer .Data represents mean ± S.D. from three independent experiments. *, *p* < 0.05; **, *p* < 0.01; ***, *p* < 0.001

The biogenesis of circRNA can be regulated in both the transcriptional and posttranscriptional levels [36, 37]. Since circPDE5A was derived from the PDE5A pre-mRNA, we first analyzed the expression level of PDE5A mRNA in the TCGA database and in PCa tissues. The results revealed that the expression of PDE5A mRNA was both downregulated both in our PCa cohort and TCGA database (Supplementary Fig. 7A, B). Since circPDE5A and PDE5A mRNA were both downregulated in PCa tissues, we hypothesized that circPDE5A and PDE5A mRNA might be regulated at the transcriptional level. So, we hypothesized if transcriptional factors, which can initiate and regulate the transcription of genes, regulated circPDE5A expression in PCa. PROMO and JASPAR databases were used to predict the transcriptional factors which might regulate PDE5A gene expression (Fig. 8A). We found six transcriptional factors (including FOXO4, CEBPB, FOXP3, SP1, CEBPA, and STAT5a) with a high possibility of binding to the promoter region (− 2000 bp to TSS) of the PDE5A gene. Since the expression level of transcriptional factors should be positively or negatively correlated with circPDE5A in PCa, FOXO4, CEBPB, and STAT5a were selected as the candidate transcriptional factors that might regulate circPDE5A expression according to the correlation analysis results in 30 paired PCa tissues (Supplementary Fig. 7C). We, therefore, evaluated whether FOXO4, CEBPB, or STAT5a regulated circPDE5A expression in PCa cells. The results showed that knockdown of FOXO4, rather than CEBPB or STAT5a, could significantly downregulate the expression of circPDE5A (Supplementary Fig. 7D), suggesting that FOXO4 may be the only transcriptional factor that regulates circPDE5A expression. Then, we evaluated whether FOXO4 regulates both circPDE5A and PDE5A mRNA expression. The FOXO4 knockdown and overexpression efficiency was detected and shown in Supplementary Fig. 7E, F. The RT-qPCR assay showed that silencing of FOXO4 decreased the expression of circPDE5A and PDE5A mRNA significantly (Fig. 8B and Supplementary Fig. 7G), while FOXO4 overexpression increased circPDE5A and PDE5A mRNA expression (Fig. 8C and Supplementary Fig. 7H). Furthermore, by analyzing the promoter sequence of PDE5A, three potential FOXO4-binding motifs were found (Fig. 8D). CHIP-qPCR analysis in 22Rv-1 cells using the FOXO4 antibody showed that FOXO4 could bind directly to the PDE5A promoter (Fig. 8D). The dual-luciferase reporter assay revealed silencing of FOXO4 inhibited PDE5A promoter activity in C4-2B and 22Rv-1 cells (Fig. 8E). We then analyzed the FOXO4 expression in the SRRSH PCa cohort and TCGA database; the results showed that FOXO4 was downregulated in PCa tissues compared to paired normal specimens (Fig. 8F and Supplementary Fig. 7I). Correlation analysis of circPDE5A and FOXO4 in the PCa cohort demonstrated the positive correlation between the circPDE5A and FOXO4 (Fig. 8G). Collectively, these data show that FOXO4 transcriptionally regulated circPDE5A in PCa cells.

### eIF4A3 regulates the expression of circPDE5A in prostate cancer

Previous studies showed that some RNA binding proteins could regulate the generation of circRNAs via binding to the circRNAs flanking intron regions [38, 39]. Using the online database Circinteractome (https://circinteractome.nia.nih.gov/), we found that eIF4A3 could specifically bind to the flanking regions of PDE5A (Supplementary Fig. 8A). We firstly verified the overexpression or knockdown efficiency of eIF4A3 in C4-2B and 22Rv-1 cells (Supplementary Fig. 8B). The RIP-qPCR assay confirmed that eIF4A3 could directly bind to the flanking region of PDE5A (Supplementary Fig. 8C). Moreover, overexpression of eIF4A3 increased circPDE5A expression, while silencing of eIF4A3 reduced the circPDE5A expression in C4-2B and 22Rv-1 cells significantly (Supplementary Fig. 8D, E). These results demonstrate that eIF4A3 regulates circPDE5A biogenesis in PCa.

## Discussion

In the current study, we profiled circRNAs expression in five paired prostate cancer and adjacent normal tissues using the circRNA array. Further validation assays showed that circPDE5A was downregulated in PCa tissues compared to normal tissues. The functional assay revealed that circPDE5A act as a metastasis suppressor that restrains PCa cells metastasis both in vitro and in vivo. Mechanistically, circPDE5A downregulated the m^6^A modification level of EIF3C mRNA by binding to WTAP and interfering with its m^6^A methylation activity. Furthermore, the reduction of the m^6^A level of EIF3C mRNA decreased the translational output in a YTHDF1-dependent manner, which suppressed the activation of the MAPK pathway.

Previous reports have illustrated that many circRNAs were abundantly and differentially expressed in various cancers [40–42]. Li et al. reported that circACC1 promoted glycolysis and fatty oxidation via stabilizing and enhancing the enzymatic activity of AMPK holoenzyme [43]. Another group revealed that circNSUN2 stabilized HMGA2 mRNA to promote colorectal liver metastasis by forming the circNSUN2/IGF2BP2/HMGA2 complex [26]. circRNAs also play a vital role in prostate cancer. hsa_circ_0001747 was reported to be a promising prognostic factor for experiencing biochemical recurrence in patients with prostate cancer [44]. circSOBP functioned as a miR-141-3p sponge to regulate amoeboid migration in prostate cancer [45]. Therefore, our study firstly performed a circRNAs array to distinguish differentially expressed circRNAs in five paired prostate cancer specimens. We identified circPDE5A as a downregulated circRNAs in prostate cancer, which have been verified in 50 paired prostate cancer and adjacent normal tissues. Functional assay confirmed that circPDE5A inhibited migration and invasion of PCa cells in vitro and in vivo.

Generally, circRNAs can regulate gene expression at the transcriptional and posttranscriptional levels. The most common role of circRNAs is binding and blocking with target miRNAs [15, 44]. Other circRNAs could interact with functional proteins [26, 40], or have protein-coding potential [46, 47]. In this study, RNA-pulldown assay was performed, and WTAP was identified to bind with circPDE5A. Intriguingly, the circPDE5A-WTAP complex only affects the N6-methyladenisine methylation activity of WTAP, while it did not change the circPDE5A or WTAP expression. m^6^A sequencing was performed, and EIF3C was chosen as one of the downstream targets of circPDE5A. Further study showed that circPDE5A regulated EIF3C expression through WTAP-dependent N6-methyladenisine methylation of EIF3C mRNA.

The eukaryotic translation initiation factor 3 (EIF3) is the largest initial factor with vital roles in translation initiation [48, 49]. Among the 13 subunits of EIF3 (EIF3A to EIF3M), EIF3C is the one of the functional components [50]. EIF3C was extensively reported involved in the progression of lung cancer, glioma, breast cancer, and ovarian cancer [30–32, 34]. However, the role of EIF3C in prostate cancer has not been discussed. In our study, EIF3C was found upregulated in PCa tissues at both the mRNA and protein levels. Transwell assay was performed, and the results showed that EIF3C could promote PCa cells migration and invasion. Next, we hypothesized how EIF3C regulated tumor progression. Zhao et al. reported that silencing of EIF3C downregulated the phosphorylation level of AKT, p38 and ERK1/2 [34], suggesting the MAPK signaling pathway is triggered by manipulating the expression of EIF3C. In our study, circPDE5A and EIF3C were both confirmed to regulate the MAPK signaling pathway, and silencing EIF3C could reverse the effect of circPDE5A on the MAPK signaling pathway.

Transcriptional factor FOXO4 has been reported to regulate various genes involved in cell cycle, apoptosis, metastasis, and metabolism in cancer cells [51]. Aberrant expression of FOXO4 is correlated with prognosis of various types of malignancy. In prostate cancer, FOXO4 was identified as a metastasis suppressor gene without affecting prostate cancer proliferation [52]. In our study, FOXO4 was identified binding to the promotor region of PDE5A using CHIP assay and dual luciferase reporter assay. What’s more, silencing of FOXO4 decreased the expression of circPDE5A, a metastasis suppressor circRNA, while overexpression of FOXO4 increased the expression of circPDE5A. Correlation analysis showed that FOXO4 and circPDE5A were positively correlated in prostate cancer tissues.

## Conclusions

In conclusion, we verified that circPDE5A exhibited a significant role in PCa cancer metastasis. circPDE5A blocks the N6-methyladenisine methylation activity of WTAP, thus decreasing the overall m^6^A level. Moreover, circPDE5A was proven to decrease the m^6^A level of EIF3C mRNA in a WTAP-dependent manner and further impaired the translational efficiency of EIF3C mRNA. circPDE5A could be regulated by transcriptional factor FOXO4 and RNA-binding protein eIF4A3. Overall, our study revealed that novel circPDE5A impaired the metastatic ability of PCa cells via the WTAP/EIF3C/MAPK pathway (Fig. 8H).

## Supplementary Information

**Additional file 1.**

**Additional file 2.**

**Additional file 3.**

**Additional file 4.**

## Data Availability

The datasets used and/or analyzed during the current study are available within the manuscript and its supplementary information files.

## Abbreviations

circRNA: Circular RNA
PCa: Prostate cancer
circPDE5A: hsa_circ_0002474
ChIP: Chromatin immunoprecipitation assay
RIP: RNA Immunoprecipitation assays
mHSPC: Metastatic hormone-sensitive prostate cancer
mCRPC: Metastatic castration-resistant prostate cancer
ncRNAs: Noncoding RNAs
RBPs: RNA binding proteins
ADT: Androgen deprivation therapy
MeRIP-seq: M^6^A methylated RNA immunoprecipitation sequencing
