# Supplementary material for: circPDE5A regulates prostate cancer metastasis via controlling WTAP-dependent N6-methyladenisine methylation of EIF3C mRNA
Source: J Exp Clin Cancer Res. 2022 Jun 2;41:187. doi: 10.1186/s13046-022-02391-5 (PMC9161465; doi:10.1186/s13046-022-02391-5)
Supplement: Supplementary file 2 — Additional file 2. [file 13046_2022_2391_MOESM2_ESM.docx]

Primers used for quantitative Real Time-PCR in this study.

| Gene Symbol | Forward primer (5’→3’) | Reverse primer (5’→3’) |
| --- | --- | --- |
| *circPDE5A* | *AGAAGTTGACCAAATTACAGGCT* | *CCATGCATTGACCATTTCTCTCT* |
| *PDE5A* | *GATCCTCGGTTCAATGCAGAA* | *ACAAAATGCCAAATAAGCAGCAA* |
| *FOXO4* | *GGCTGCCGCGATCATAGAC* | *GGCTGGTTAGCGATCTCTGG* |
| *eIF4A3* | *AAGGGAGAGATGTCATCGCAC* | *GCTTGAGTTTCACGAACCTGA* |
| *WTAP* | *CTTCCCAAGAAGGTTCGATTGA* | *TCAGACTCTCTTAGGCCAGTTAC* |
| *GPSM2* | *TGCAAGGACTATTGGAGACCA* | *TCTGGAAATATCTAGGTGTCGCT* |
| *AL359091* | *AGTCGGCACAGAGGAAGG* | *GCACAGGCAGGCAGACAT* |
| *CXorf49* | *TGTCCTCGGTGCATCTGG* | *TTGCTTTGAACTGCGTTGG* |
| *PERM1* | *TGCCATCCGCTACTTCCG* | *CTGTGGTCGTCTCCTCATCC* |
| *LNPEP* | *ACCAGATGTGGTGGATTTAGCC* | *CAGTTGCACTGTTCCGAAGG* |
| *PAG1* | *TTCCTGTGCTCTAGTTGTGACA* | *CACGTTCATCAGGTTCTCATGG* |
| *EIF3C* | *ATGTCGCGGTTTTTCACCAC* | *GCGGACAACTCTCTTGGTATCT* |
| *CCZ1* | *ACAAGGACATTTAGCCCATCAAA* | *GATTCCGAACAACCATGACCA* |
| *SMN1* | *CCTGTGTTGTGGTTTACACTGG* | *GGGGGAATTATCTTTCCTGGTCC* |
| *POLR2J2* | *TGCTGGCTACAAAGTCCC* | *TGATGGCGTTGGTAAAGG* |
| *ABHD17A* | *GCGTCGCTTCATCTCCCA* | *TGCGTAGCTCTGTTGCCTGTA* |
| *AP000640* | *CGGCTCCCATAAAGATAC* | *ACCCTTGATTCCTGCTGT* |
| *TNS1* | *GTACGTCACAGAGAGGATCATCG* | *GCAGGTAGTTGCCTCCATGTT* |
| *GSTT4* | *GGTCACCACCACAGCAAA* | *CCAAGCCACGAACTCATC* |
| *TMEM259* | *CGAGACGCCCACCAAAGT* | *CATCGTAGCCCAGGAACTCAT* |
| *PLXNA3* | *AGTCCTGCTATCGTGGGGAG* | *CAGAAGTTGCCGTTGATCTGC* |
| *GAPDH* | *CAAGGTCATCCATGACAACTTTG* | *GTCCACCACCCTGTTGCTGTAG* |
| Primers used for CHIP-qPCR |  |  |
| Gene Symbol | Forward primer (5’→3’) | Reverse primer (5’→3’) |
| *PDE5A#1* | *AGGTGCCCTTGTTTTCTA* | *GCCTCAAACTTCCCTTAT* |
| *PDE5A#2* | *CCCGACCCTCTTGGTATT* | *TTGCTGATTGGATTTTGG* |
| *PDE5A#3* | *GGGACAGCCCAAAGGCAACA* | *GGACAAGCAGGAGACTGGAAGG* |

siRNAs used in this study.

| Gene Symbol | siRNA sequence (sense 5’→3’) |
| --- | --- |
| *si-circPDE5A#1* | *GGAAGAGAGAAATGGTCAA* |
| *si-circPDE5A#2* | *AATCATAGGGAAGAGAGAA* |
| *si-FOXO4#1* | *UCAGAAAGUCGCGAACUUCCC* |
| *si-FOXO4#2* | *UUGAAGUAGGGUACAGUACGG* |
| *si-eIF4A3* | *AGAUCAAAGCUUGAGUUUCAC* |
| *si-WTAP* | *CACAGAUCUUAACUCUAAUTT* |
| *si-YTHDF1* | *GUGGGACAAAUGUGAACAUTT* |
| *si-EIF3C#1* | *GGCCUCAAGAUUUCUUAAATT* |
| *si-EIF3C#2* | *GAGGCAACUAUGGCAAACATT* |

Antibody used in this study

| Antibody | company | catalog number |
| --- | --- | --- |
| *WTAP* | *Proteintech* | *60188-1-Ig* |
| *GAPDH* | *abcam* | *ab8245* |
| *Histone H3* | *abcam* | *ab1791* |
| *FOXO4* | *abcam* | *ab128908* |
| *EIF4A3* | *proteintech* | *17504-1-AP* |
| *m^6^A* | *abcam* | *ab258077* |
| *METTL3* | *abcam* | *ab195352* |
| *METTL14* | *abcam* | *ab220030* |
| *EIF3C* | *Proteintech* | *12733-1-AP* |
| *FLAG* | *sigma* | *F1804* |
| *YTHDF1* | *Proteintech* | *17479-1-AP* |
| *AKT* | *CST* | *4685* |
| *phospho-AKT* | *CST* | *4060* |
| *P38* | *CST* | *9212s* |
| *p-P38* | *CST* | *9215s* |
| *mTOR* | *CST* | *2983* |
| *p-mTOR* | *CST* | *5536s* |

Biotinylated probe and sequence

| probe | sequence |
| --- | --- |
| *circPDE5A* | *5' biotin-aaaTGCATTGACCATTTCTCTCTTCCCTATGATTC 3’* |
| *control probe* | *5' biotin-aaaGCAGCCTGATCACGACTGACTTTAGTGTTTGCATT 3’* |

FISH oligonucleotide probe

| probe | sequence |
| --- | --- |
| *circPDE5A* | *5' CY3-CCAT+TTCTCTCT+TCCCTATGAT+TCT 3’* |
