# Supplementary material for: circPDE5A regulates prostate cancer metastasis via controlling WTAP-dependent N6-methyladenisine methylation of EIF3C mRNA
Source: J Exp Clin Cancer Res. 2022 Jun 2;41:187. doi: 10.1186/s13046-022-02391-5 (PMC9161465; doi:10.1186/s13046-022-02391-5)
Supplement: Supplementary file 4 — Additional file 4. [file 13046_2022_2391_MOESM4_ESM.docx]

**Supplementary figures and figure legends:**

**
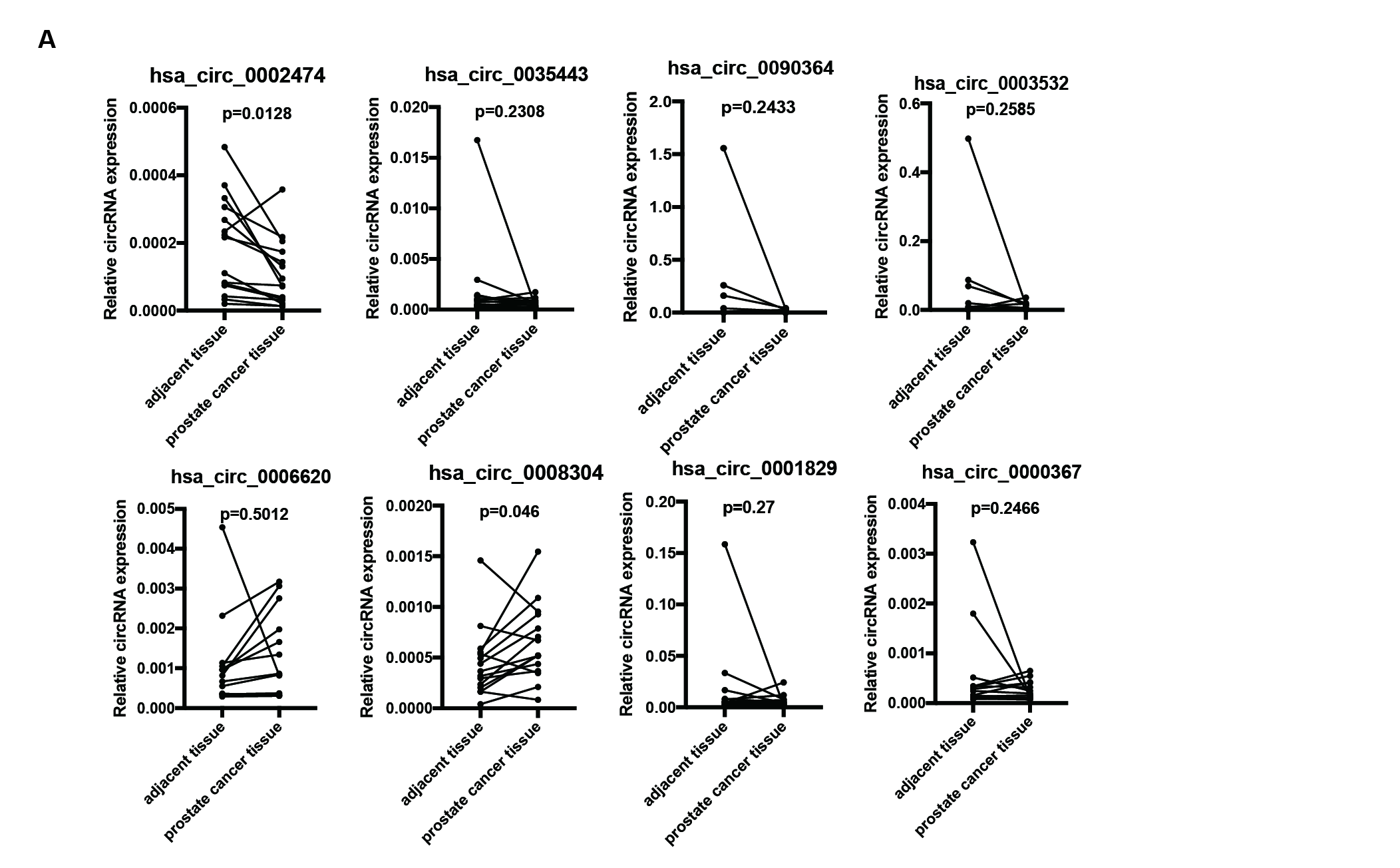
**

**Supplementary Figure 1. A,** Relative expression levels of 8 candidate circRNAs in 15 paired prostate cancer and adjacent normal tissues.

**
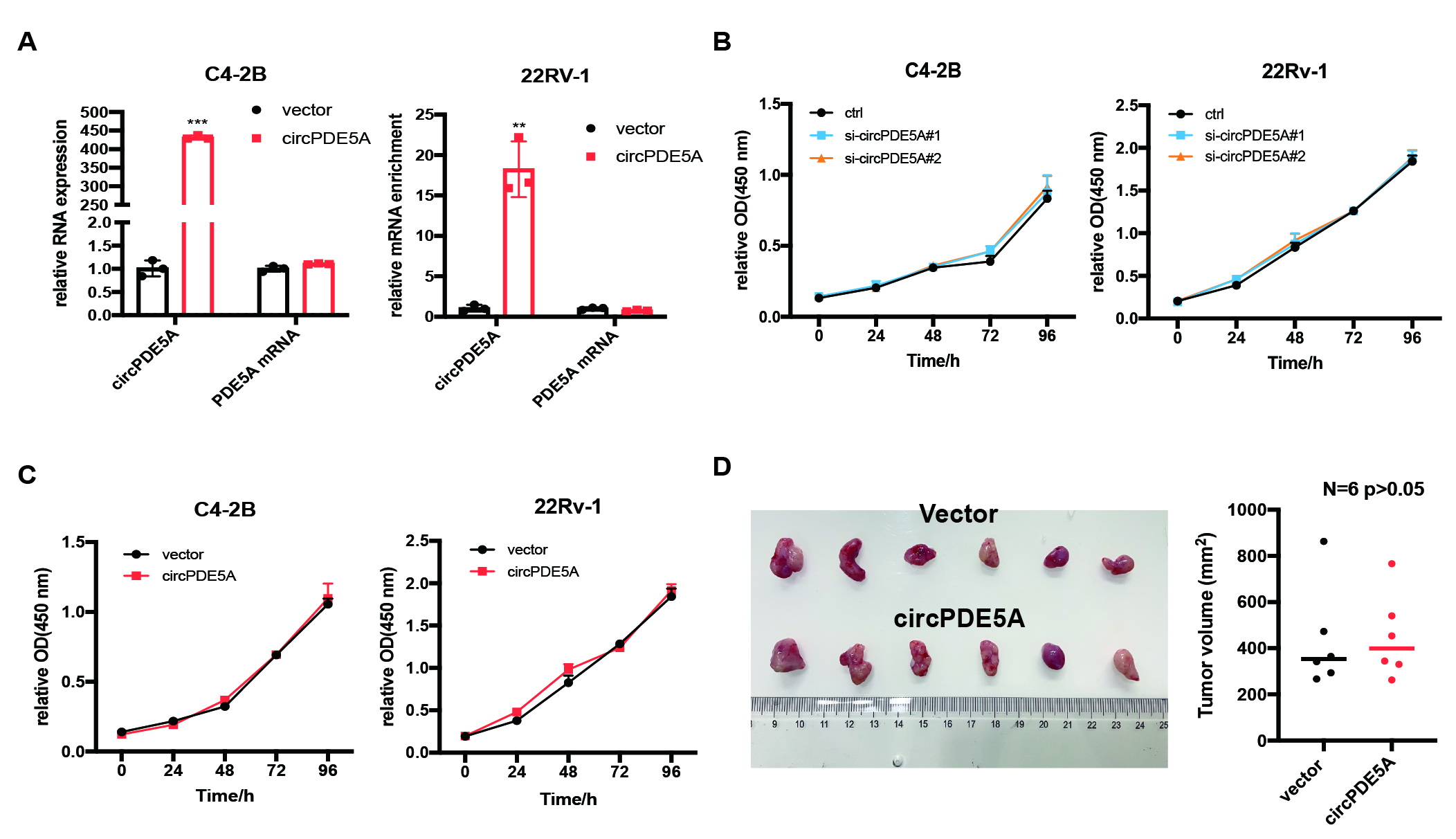
**

**Supplementary Figure 2. A,** qRT-PCR assay showing the circPDE5A overexpression efficiency. **B,** CCK-8 assay in C4-2B and 22Rv-1 cells transfected with circDPE5A siRNAs. **C,** CCK-8 assay in C4-2B and 22Rv-1 cells transfected with circDPE5A overexpression plasmids. **D,** A xenograft animal model showing the effect of circPDE5A overexpression on 22Rv-1 cell’s proliferation. Data represents mean±S.D. from three independent experiments. **, *p* < 0.01; ***, *p* < 0.001.

**
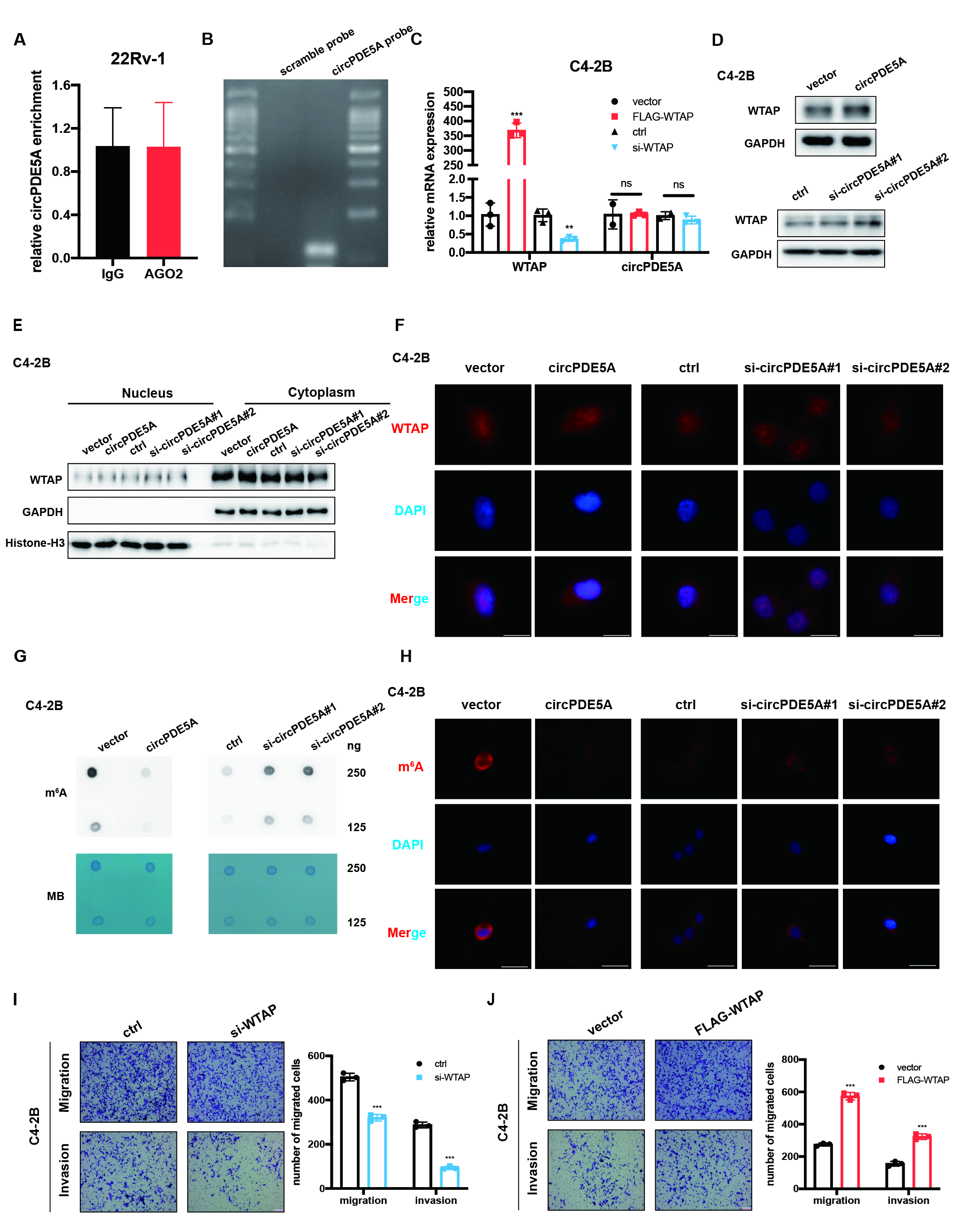
**

**Supplementary Figure 3. A,** AGO2-RIP assay showing the binding capacity between circPDE5A and AGO2 in 22Rv-1 cells. **B,** DNA gel suggesting the specificity of the circPDE5A probe. **C,** Analysis of the expression of circPDE5A in C4-2B cells with WTAP overexpression or knockdown through RT-qPCR. **D,** Analysis of the expression of WTAP in C4-2B cells with circPDE5A overexpression or knockdown through western blotting. **E,** The cellular distribution of WTAP in C4-2B cells with circPDE5A overexpression or knockdown through western blotting. **F,** Analysis of the cellular distribution of WTAP in C4-2B cells with circPDE5A overexpression or knockdown through immunofluorescence. Scale bars, 10μm. **G,** Dot blot showing the total m^6^A modification level in C4-2B cells with circPDE5A overexpression or knockdown. **H,** Immunofluorescence showing the total m^6^A modification level in C4-2B cells with circPDE5A overexpression or knockdown. Scale bars, 10μm. **I, J,** Transwell assay showing the migration and invasion ability with WTAP knockdown (**I**) or overexpression (**J**) in C4-2B cells. Scale bars, 5μm. Data represents mean±S.D. from three independent experiments. **, p < 0.01; ***, p < 0.001.

**
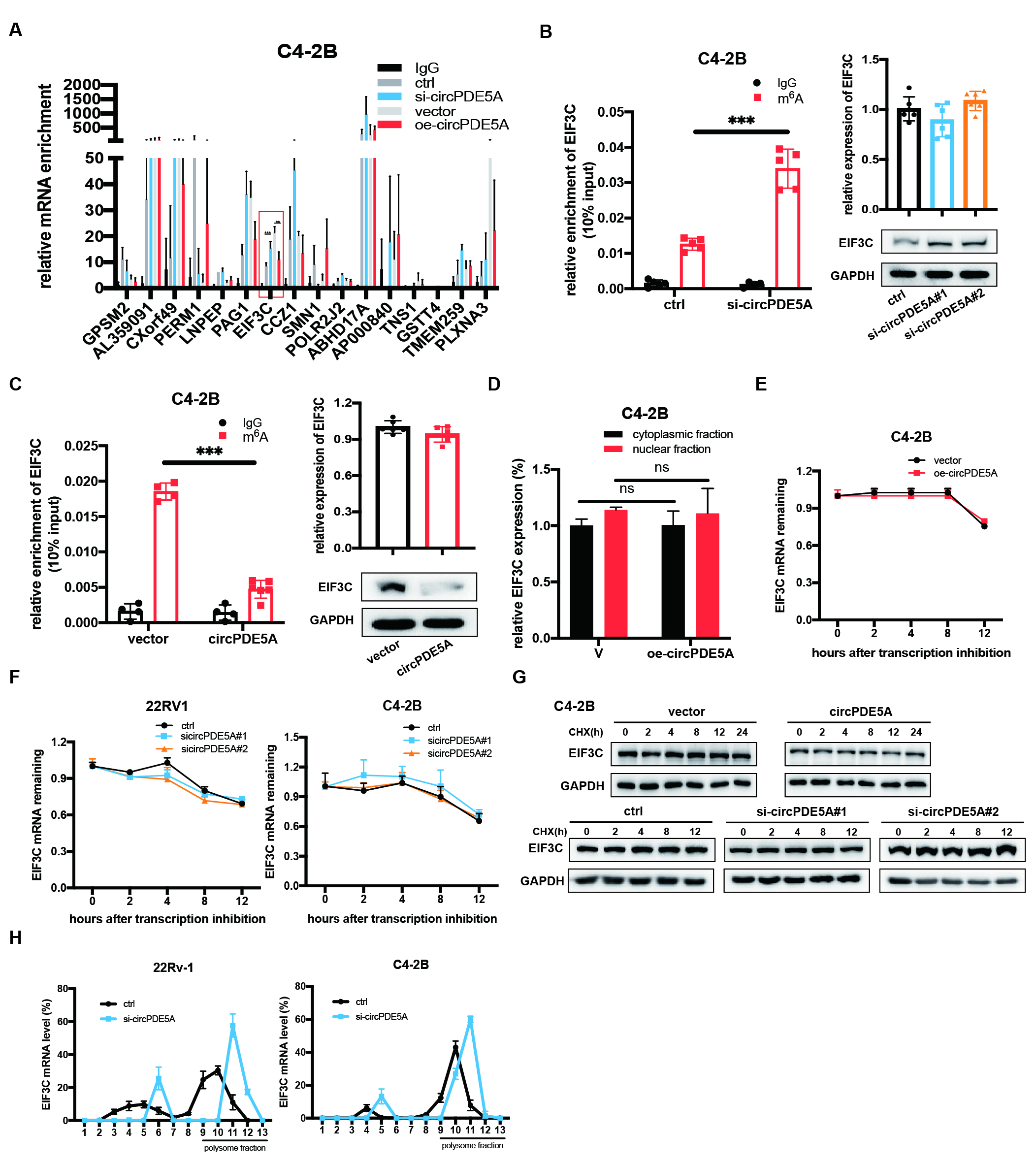
**

**Supplementary Figure 4. A,** MeRIP assay showing the m^6^A modification level of 16 genes in C4-2B cells with circPDE5A knockdown or overexpression. **B, C,** Left: MeRIP assay showing the m^6^A modification level of EIF3C in C4-2B cells with circPDE5A knockdown (**B**) or overexpression (**C**); right: the mRNA and protein expression of EIF3C in C4-2B cells with circPDE5A knockdown (**B**) or overexpression (**C**). **D,** RT-qPCR assay showing the cellular distribution of EIF3C in C4-2B cells with circPDE5A overexpression. **E,** RT-qPCR assay showing the EIF3C mRNA stability in C4-2B cells with circPDE5A overexpression. **F,** RT-qPCR assay showing the stability of EIF3C mRNA in 22Rv-1 and C4-2B cells with circPDE5A knockdown. **G,** WB assay showing the stability of EIF3C protein in C4-2B cells with circPDE5A overexpression or knockdown. **H,** RT-qPCR assay showing the relative level of EIF3C mRNA in gradient fractions with circPDE5A knockdown in C4-2B and 22Rv-1 cells. Data represents mean±S.D. from three independent experiments. **, *p* < 0.01; ***, *p* < 0.001.

**
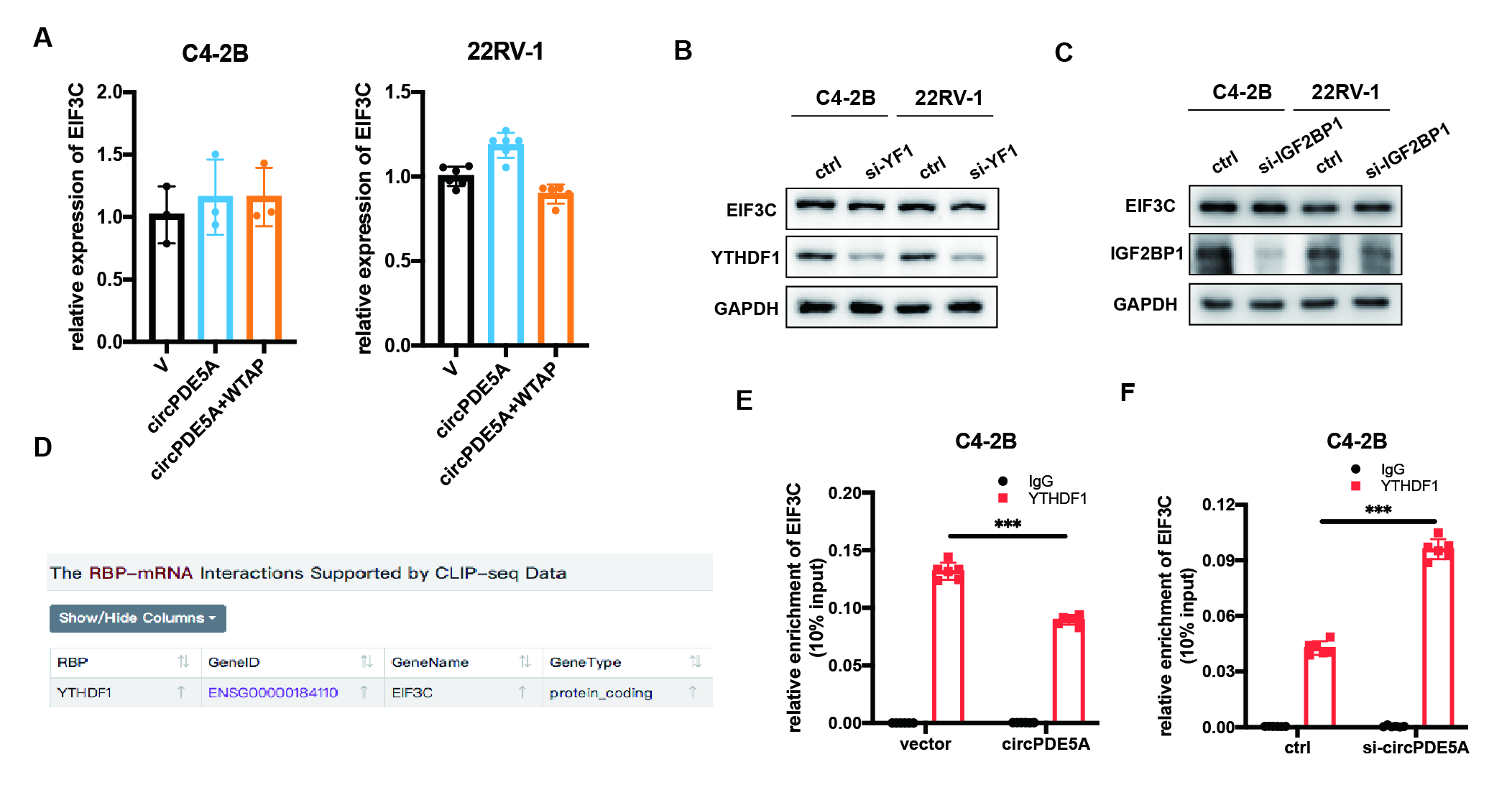
**

**Supplementary Figure 5. A,** RT-qPCR assay showing the mRNA levels of EIF3C in circPDE5A overexpression C4-2B and 22Rv-1 cells with the overexpression of WTAP. **B,** Western blotting assay showing the protein level of EIF3C and YTHDF1 in C4-2B and 22Rv-1 cells with YTHDF1 knockdown. **C,** Western blotting assay showing the protein level of EIF3C and IGF2BP1 in C4-2B and 22Rv-1 cells with IGF2BP1 knockdown. **D,** The potential interaction between YTHDF1 and EIF3C mRNA according to the CLIP-seq data from the Starbase database. **E, F,** YTHDF1-RIP assay showing the binding capacity between YTHDF1 and EIF3C mRNA in C4-2B cells with circPDE5A knockdown (**E**) or overexpression (**F**). Data represents mean±S.D. from three independent experiments. ***, *p* < 0.001.

**
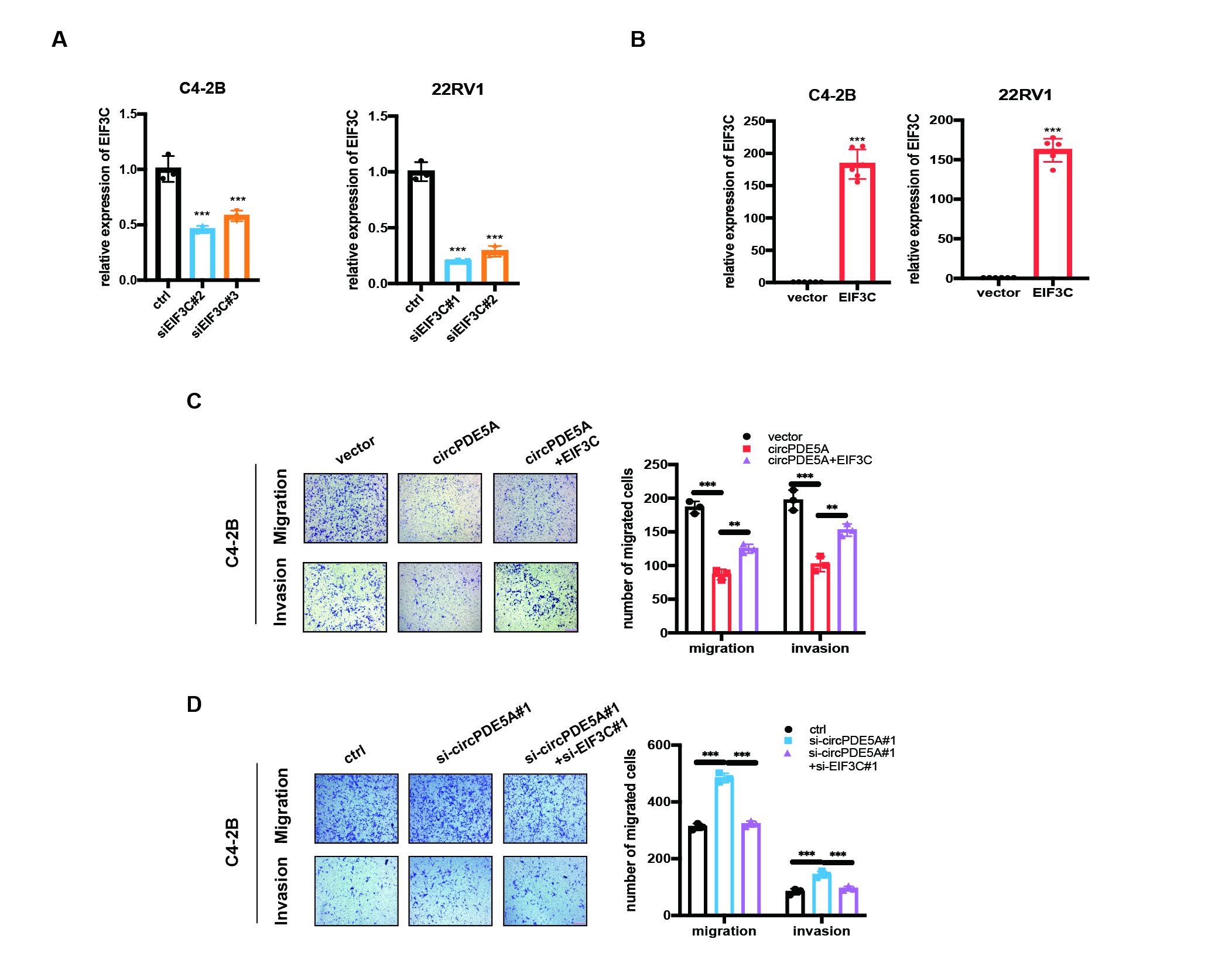
**

**Supplementary Figure 6. A,** RT-qPCR showing the knockdown efficiency of EIF3C in C4-2B and 22Rv-1 cells. **B,** RT-qPCR showing the overexpression efficiency of EIF3C in C4-2B and 22Rv-1 cells. **C,** Transwell assay showing the migration and invasion ability of circPDE5A overexpression C4-2B cells with EIF3C overexpression. Scale bars, 5μm. **D,** Transwell assay showing the migration and invasion ability of circPDE5A knockdown 22Rv-1 cells with EIF3C knockdown. Scale bars, 5μm. Data represents mean±S.D. from three independent experiments. **, *p* < 0.01; ***, *p* < 0.001.

**
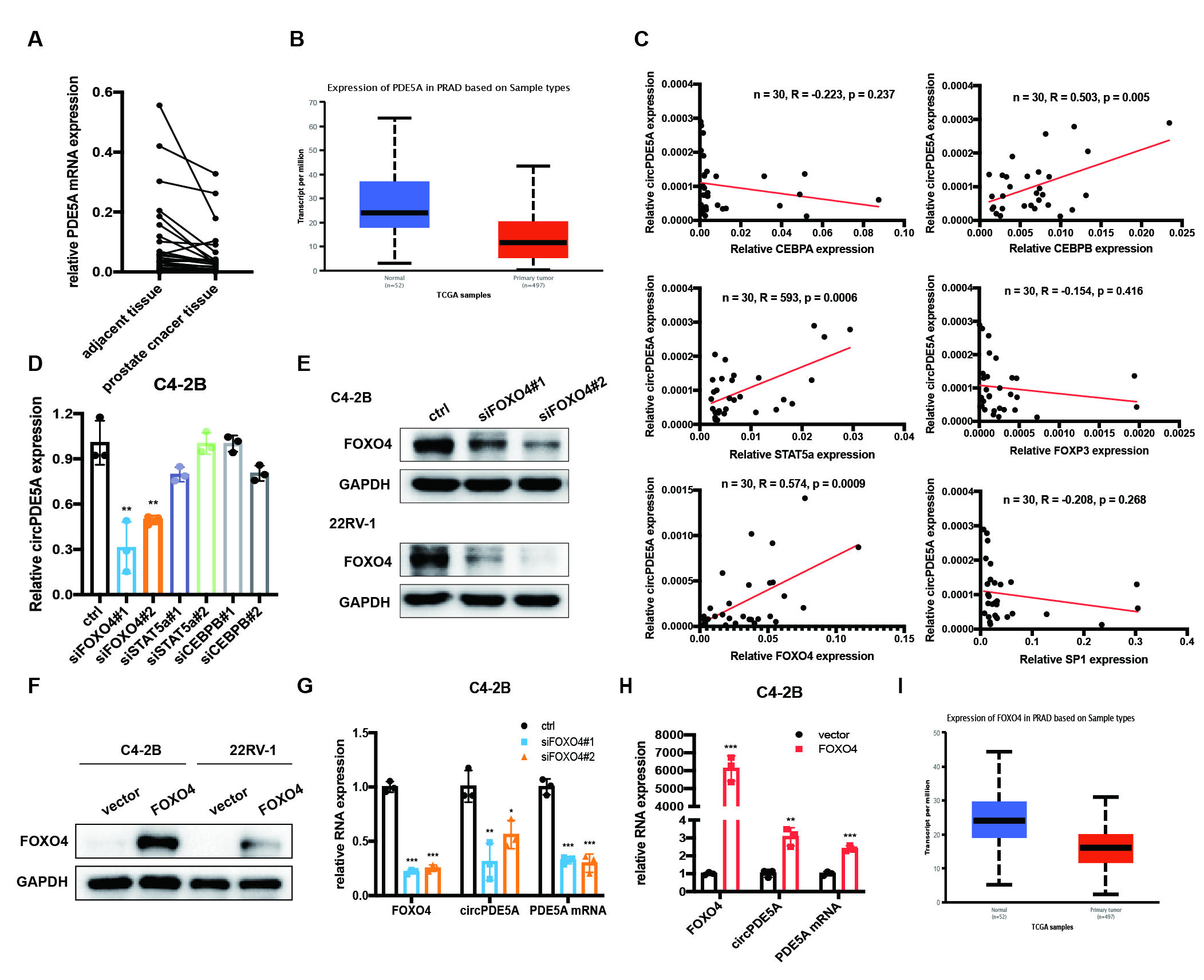
**

**Supplementary Figure 7. A,** Relative expression levels of PDE5A mRNA in 50 paired prostate cancer tissues and adjacent normal tissues. **B,** Relative expression levels of PDE5A mRNA in TCGA database (PRAD). **C,** Correlation analysis of the expression between CEBPA, CEBPB, STAT5a, FOXP3, FOXO4, SP1 and circPDE5A in 30 paired PCa specimens. **D,** Relative expression levels of circPDE5A in FOXO4, STAT5a and CEBPB knockdown C4-2B cells. **E,** Western blotting assay showing the knockdown efficiency of FOXO4 in C4-2B and 22Rv-1 cells. **F,** Western blotting assay showing the overexpression efficiency of FOXO4 in C4-2B and 22Rv-1 cells. **G,** The expression of circPDE5A and PDE5A mRNA in FOXO4 knockdown C4-2B cells. **H,** The expression of circPDE5A and PDE5A mRNA in FOXO4 overexpression C4-2B cells. **I,** Relative expression level of FOXO4 mRNA in TCGA database (PRAD). Data represents mean±S.D. from three independent experiments. *, *p* < 0.05; **, *p* < 0.01; ***, *p* < 0.001.

**
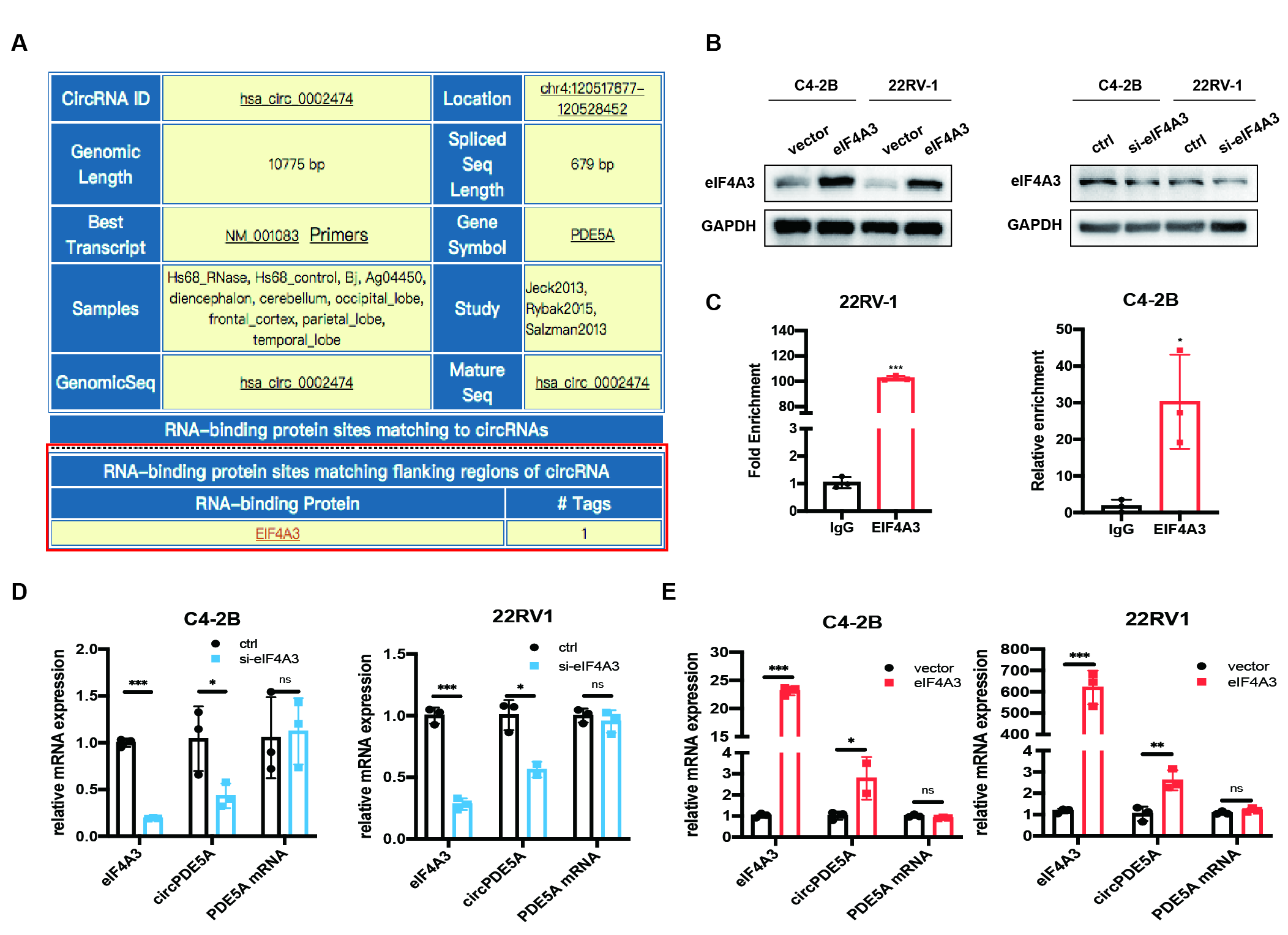
**

**Supplementary Figure 8. A,** Potential RNA-binding protein sites matching that of circPDE5A according to Circinteractome. **B,** Western blotting assay showing the knockdown or overexpression efficiency of eIF4A3 in C4-2B and 22Rv-1 cells. **C,** eIF4A3-RNA-IP assay showed the binding capacity between circPDE5A and EIF4A3. **D, E** The expression of circPDE5A and PDE5A mRNA in eIF4A3 knockdown or overexpression 22Rv1 and C4-2B cells. Data represents mean±S.D. from three independent experiments. *, *p* < 0.05; ***, *p* < 0.001.
